# Rational engineering of a mesohalophilic carbonic anhydrase to an extreme halotolerant biocatalyst

**DOI:** 10.1038/ncomms10278

**Published:** 2015-12-21

**Authors:** Andrew C. Warden, Michelle Williams, Thomas S. Peat, Shane A. Seabrook, Janet Newman, Greg Dojchinov, Victoria S. Haritos

**Affiliations:** 1 Energy Flagship, Commonwealth Scientific and Industrial Research Organisation (CSIRO), GPO Box 1700, Canberra, Australian Capital Territory 2601, Australia; 2 Land and Water Flagship, Commonwealth Scientific and Industrial Research Organisation (CSIRO), GPO Box 1700, Canberra, Australian Capital Territory 2601, Australia; 3 Biomedical Manufacturing Program, Commonwealth Scientific and Industrial Research Organisation (CSIRO), 343 Royal Parade, Parkville, Victoria 3052, Australia; 4 Department of Chemical Engineering, Monash University, Clayton, Victoria 3168, Australia

## Abstract

Enzymes expressed by highly salt-tolerant organisms show many modifications compared with salt-affected counterparts including biased amino acid and lower α-helix content, lower solvent accessibility and negative surface charge. Here, we show that halotolerance can be generated in an enzyme solely by modifying surface residues. Rational design of carbonic anhydrase II is undertaken in three stages replacing 18 residues in total, crystal structures confirm changes are confined to surface residues. Catalytic activities and thermal unfolding temperatures of the designed enzymes increase at high salt concentrations demonstrating their shift to halotolerance, whereas the opposite response is found in the wild-type enzyme. Molecular dynamics calculations reveal a key role for sodium ions in increasing halotolerant enzyme stability largely through interactions with the highly ordered first Na^+^ hydration shell. For the first time, an approach to generate extreme halotolerance, a trait with broad application in industrial biocatalysis, in a wild-type enzyme is demonstrated.

## Abstract (web summary)

Halophilic organisms thrive in high salt conditions and express proteins that display desirable characteristics for industrial applications. Here, the authors use a rational design approach to transform wild-type carbonic anhydrase into a strongly halophilic enzyme.

## Graphical abstract

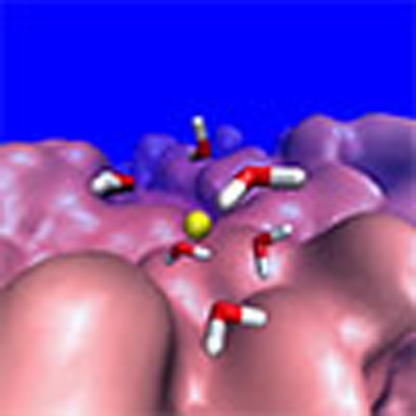

For many enzymes, an optimal operating environment is characterized by moderate temperature, pH and salinity range and moving outside of these conditions can lead to rapid denaturation of the protein. In particular, hypersaline conditions can cause aggregation of mesohalophilic enzymes, that is, salt-sensitive, due to an increase in the hydrophobic effect and structural collapse as the high concentration of ions interferes with electrostatic interactions between amino acid residues^1^. There are, however, halophilic enzymes that have been isolated from organisms living in high-salt environments, such as the Dead Sea^2^, which are stable and perform optimally in these conditions.

Compared with their non-halophile counterparts, halophilic enzymes typically have significantly higher densities of negative charge on their surfaces and reduced levels of lysine and cysteine residues but higher amounts of random coil structure at the expense of α-helix, higher content of aspartate and small hydrophobic residues^3^,^4^,^5^,^6^. The overall reduction in size of hydrophobic residues in halophilic proteins is thought to be a compensation for an increase in the hydrophobic effect brought about by the increase in the dielectric character of high salt concentrations. Conversely, enzymes from halophilic organisms that show adaptations to high salt conditions do not easily express and fold in typical recombinant systems and are highly unstable in low-salt conditions^2^.

Industrial use of biocatalysts spans from fine chemical and polymer manufacturing^7^, via intermediates and has broadened into energy with biofuels^8^, CO_2_ capture^9^ and fuel cells^10^ among others^11^. Halophilic enzymes have received growing attention as biocatalysts in hypersaline environments such as brines, ionic liquids (ILs), ionic detergents and where solutes are formed by gases dissolving into aqueous systems^12^. Sourcing of enzymes for use in high-salt conditions has traditionally been through discovery of halophilic enzymes from natural systems, but this approach can meet significant hurdles in being able to achieve, for example, recombinant protein expression, folding and stability or even the desired catalytic activity. An alternative approach to biological discovery is to rationally design halotolerance into an existing biocatalyst that already possesses the desired attributes of catalytic activity, facile heterologous expression, tolerance of inhibitory chemicals or other essential characteristics.

Recent reports of enzyme engineering that focussed specifically on halotolerance include the characterization of the effect of two amino acid changes on the halophilicity of dinucleotide diphosphate kinase^13^, and a more extensive undertaking that examined the structural basis and amino acid content determining salt tolerance of the 1A domain of NAD^+^-dependent DNA ligase from *Haloferax volcanii* and the IgG-binding domain of Protein L from *Streptococcus magmus*^14^. However, these approaches have generally started with knowledge of the halotolerant protein and structure and have worked backwards to the non-tolerant protein.

Thus, to better understand the key factors controlling halophilicity in enzymes and to develop a halotolerant biocatalyst for hypersaline conditions, we undertook rational enzyme design by selecting just one of several mechanisms present in halophilic enzymes: increasing the incidence of acidic residues on the enzyme surface. A well-studied carbonic anhydrase II was selected for rational design as it has efficient CO_2_ hydration activity and could be heterologously expressed in *Escherichia coli*. Herein, we describe for the first time the successful redesign of a mesohalophile enzyme to an extremely halotolerant orthologue, that is, one that is active at 3 M NaCl and above, by altering 18 surface residues. The conversion from mesohalophile to extreme halotolerant enzyme is conducted in stages to assess the effects of substitutions on overall protein stability and degree of halotolerance. Furthermore, molecular dynamics calculations suggest that it is both extended binding of cations by acidic residues with complementary chelating partners, and interactions through the highly ordered hydration shells of the cations that are primary mechanisms of halotolerance.

## Results

### Crystal structures of mutants 1-4 (M1-M4)

Wild-type (WT) and designed M1–M4 carbonic anhydrases were efficiently expressed in *E. coli* and purified as folded and functional enzymes using standard procedures (Supplementary Fig. 1). With the exception of N62D, all other substitutions in this study have not been previously investigated in carbonic anhydrase II. The most substituted enzyme, M4, had 10 additional Asp and eight extra Glu residues but five fewer Asn, four fewer Gln, three fewer Lys, two fewer Val and one less Gly, Leu, Arg and Thr residues compared with WT (Supplementary Table 1). Crystal structures of WT and engineered enzymes were obtained to confirm the presence of changed residues, that active site residue positioning was unaffected and also to compare the overall structural similarity of the designed proteins with that of WT. Naturally occurring halophilic enzymes are underrepresented in the protein data bank as these enzymes are generally highly soluble in ‘salting out' salts used to generate crystals of mesohalophilic enzymes^2^.

Crystal statistics for the WT and designed enzymes are given in Table 1. The structures of the four engineered enzymes superposed almost perfectly with the WT enzyme (maximum root mean square deviation (RMSD)=0.42 Å for M4) with no significant differences apparent in their secondary structures (Supplementary Fig. 2). Three of the four variants (M1, M3 and M4) included the mutation N62D, which has been previously reported^15^ and been shown to reduce the activity of human CAII attributed to the effect of holding the side chains of the catalytically important His64 in the ‘outwards' position, compared with that determined for the ‘inward' facing conformation of this residue. The crystal structures of the three N62D containing enzymes (M1, M3 and M4) also showed His64 as the outwards rotamer, whereas M2 showed it as approximately equal proportions of the inwards and outwards rotamers. Some of the orientations of other surface residues of M1–M4 were also altered compared with the WT, although none, with the exception of N62D, were in the vicinity of the active site. While there were variations in the water network among the mutants, it was difficult to make meaningful, direct comparisons owing to differences in resolution between data sets. There was extra electron density in the structure of M4, where it appeared to fit a D130E substitution, but this was not included in any of the mutants. Gene and protein analysis did not support any substitution at this position and the extra electron density remains unmodelled. Representative portions of the electron density maps for M1–M4 are shown in Supplementary Fig. 3.

### Extreme haloterance of M4 supported by thermal unfolding

The effect of added salts, ILs and the denaturants, GuHCl and urea on the thermal unfolding values of WT and M1–M4 enzymes were assessed over the pH range 7–11 using differential scanning fluorimetry (DSF). Of the buffers employed, Tris-HCl in the pH range 8–9 provided the highest thermal unfolding values (*T*_h_) and the results presented in this section pertain to experiments conducted in 50 mM Tris-HCl pH 8.5. The *T*_h_ values for the enzymes in the buffer conditions were WT 67.5 °C, M1 66.5 °C, M2 50.0 °C, M3 50.0 °C and M4 42.5 °C.

**Figure 1:**
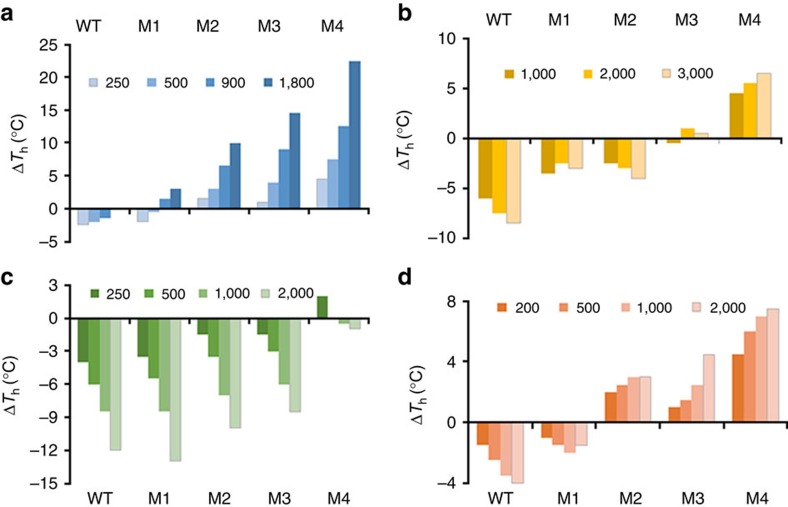
Thermal unfolding analyses of WT and M1–M4 carbonic anhydrases. Experiments were performed in the presence of increasing concentrations of (**a**) Na_2_SO_4_, (**b**) NaCl, (**c**) NaNO_3_ and (**d**) ionic liquid EtOH-AF. For each enzyme, thermostability is indicated by the difference in thermal unfolding value (*T*_h_) obtained by DSF in the presence of salt and 50 mM Tris-HCl buffer pH 8.5 versus buffer only.

The addition of Na_2_SO_4_ at all concentrations tested increased the thermal unfolding temperatures for M2–M4 with M4 most positively affected (Fig. 1a). At 1,800 mM, the unfolding temperature for M4 increased by 22.5 °C, which brought its unfolding temperature to the highest *T*_h_ observed for WT. Conversely, the unfolding temperatures for WT were reduced by up to 2 °C in the presence of Na_2_SO_4_ with M1 showing an intermediate response. The effect of NaCl on unfolding temperature was investigated in hypersaline conditions, that is, normal seawater has very ∼600 mM NaCl and the lowest concentration tested here was 1,000 mM. Similarly, addition of NaCl to the thermal unfolding analysis resulted in contrasting effects for WT and M4; the *T*_h_ for WT was reduced as the concentration of NaCl increased, whereas M4 was increasingly stabilized with increasing NaCl concentration (Fig. 1b). The unfolding temperatures for the intermediate enzymes (M1–M3) showed a transitional relationship, where M3 was unaffected by added NaCl whereas the effects on M1 and M2 were between that for WT and M3. Among the sodium salts tested, the most negative effect on unfolding temperature was caused by NaNO_3_ which substantially reduced *T*_h_ for all the carbonic anhydrases in a concentration-dependent manner except for M4 whose *T*_h_ was barely affected by the addition of the salt up to 2 M (Fig. 1c).

Four ILs were tested against the WT and M1–M4 enzymes in 50 mM Tris-HCl at pH 8.5. There was a clear difference in the effect of the addition of EtOH-AF to the enzymes; *T*_h_ values for WT and M1 were reduced with increasing IL concentration, whereas this caused an increasingly positive effect on unfolding temperature for M2 through to M4 (Fig. 1d). The effects of adding ethyl-AF, ethyl-NO3 and DMI-DMP on WT and the designed enzymes were similar, all ILs reduced *T*_h_ for WT and M1–M3 and the effect increased with increasing concentration. However, the *T*_h_ for M4 was much less affected by IL addition than the other enzymes and was even positively affected by the addition of ethyl-AF (Supplementary Fig. 4a–c).

The addition of denaturants urea and GuHCl, to enzymes in the DSF analysis affected the thermal unfolding temperatures for WT and M1–M3 enzymes similarly, that is, the change in *T*_h_ values were similarly negative for all enzymes with addition of denaturant (Supplementary Fig. 5a,b). However, the *T*_h_ for M4 was affected less by GuHCl than the other enzymes, although similarly affected by urea.

While the thermostability of M4 was increased by the addition of salts to the basic buffer condition, the unfolding temperatures of any of the carbonic anhydrases did not exceed that for native bCAII in buffer.

### Secondary structure stability is greater for M4 in SDS

**Figure 2:**
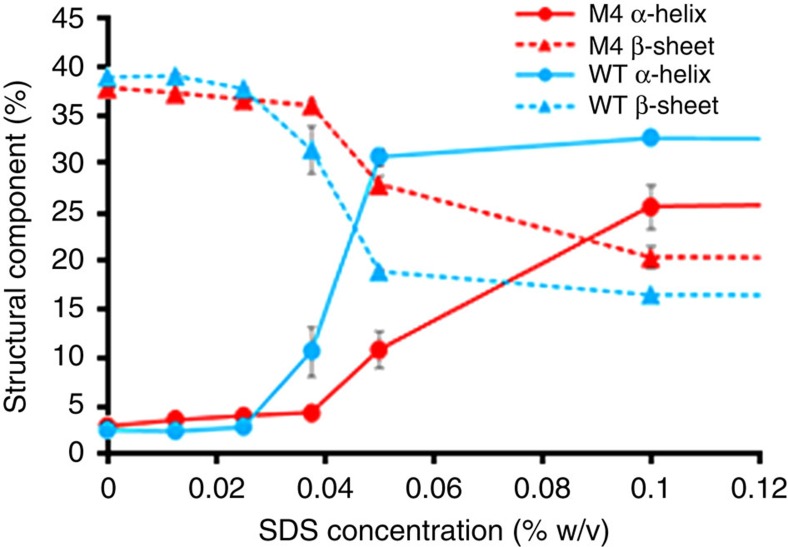
Circular dichroism analysis of WT and M4 in SDS. Mean percentage (±s.d.) α-helix and β-sheet secondary structure content in WT and M4 carbonic anhydrase with increasing concentrations of the detergent SDS (%w/v) in 10 mM Tris-HCl pH 8 as determined by circular dichroism spectroscopy.

WT was more susceptible than M4 to the secondary structure-disrupting effects of SDS as observed by circular dichroism (CD) spectroscopy (Fig. 2). Most β-sheet structure was absent and additional α-helix was formed in WT enzyme exposed to 0.0375% w/v SDS, whereas disruption to secondary structure was relatively minor in M4 at the same concentration (Fig. 2). Preincubation of the enzymes for 60 min before CD analysis yielded a very similar pattern of secondary structure disruption to that where spectra were read immediately following SDS exposure (Supplementary Fig. 6). Conversely, the loss of secondary structure in WT, M3 and M4 by the addition of GuHCl was similar at each concentration tested between 250 and 5,000 mM (Supplementary Fig. 7).

### Catalytic activity of M4 is increased in high salt

The catalytic activities of WT and engineered enzymes M1–M4 were assessed by measuring activity through standard assays in the presence and absence of a range of salts and denaturants. 4-NPA esterase catalytic rates of M1–M4 measured under standard assay conditions and low ionic strength (*I*=0.2 M) were reduced compared with WT, as shown in Table 2. Both M1 and M2 contained six substitutions with each change exclusive to each variant, but the enzymes displayed similar catalytic activities in low ionic strength conditions. However, only M1 carried the N62D substitution and its catalytic activities were 86% and 61% of the WT activity for CO_2_ hydration and esterase, respectively (Table 2). M3 and M4, having the largest numbers of amino acid changes, exhibited the lowest esterase activities under these conditions with M4 reduced by ∼66% compared with WT. Esterase activities determined over points within the pH range of 6.8–8 showed maximal activity at pH 8 for all enzymes, suggesting that there has been no change to the properties of the active nucleophile and adjacent residues (Supplementary Fig. 8).

**Figure 3:**
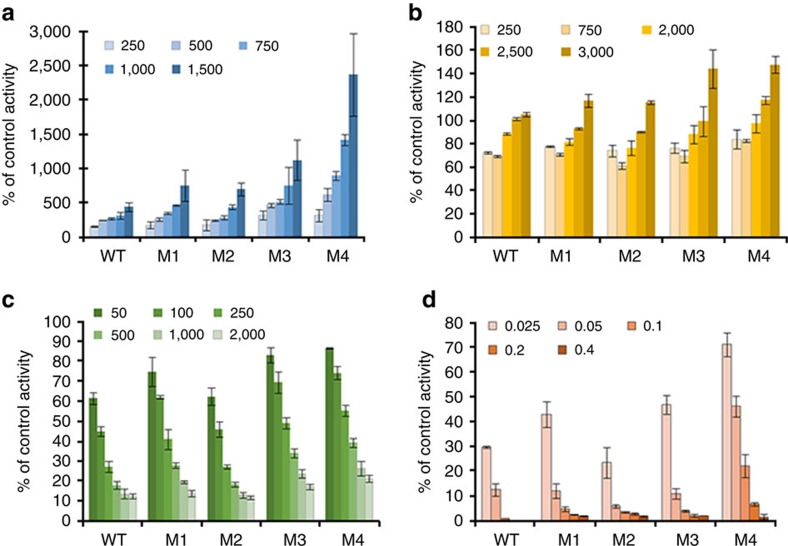
Effect of salts and denaturants on esterase activity of WT and M1–M4. Graphs indicate percentage of control activity±s.d. for enzymes in (**a**) Na_2_SO_4_, (**b**) NaCl, (**c**) NaNO_3_ and (**d**) SDS. Salts are shown as millimolar concentrations except SDS which is %w/v. Control activity refers to the activity of the individual enzyme in the absence of added salt.

The addition of sodium salts of sulfate, chloride and nitrate to the 4-NPA assay buffer had potent effects on the apparent activities. Addition of Na_2_SO_4_ over the concentration range 250–1,500 mM had a strong positive effect on the catalytic activities of M3 and M4 in particular, and these increases were significantly greater compared with the increases for WT (Fig. 3a). Esterase activity for M4 was highest where Na_2_SO_4_ was added at 1,500 mM and restored the enzyme's initial velocity to for the same as WT under identical conditions. Sodium chloride added to the assay mixture at concentrations of 750 mM and below inhibited esterase activity of all enzymes by up to 40% with M4 being the least affected (Fig. 3b). At 2,500 mM and above, the esterase activity of M1–M4 enzymes increased above activities measured in the absence of NaCl with significant increases in activity for M3 and M4 (Fig. 3b). However, there was no condition identified where the activities of M1–M4 exceeded that of WT under basic buffer conditions.

Sodium nitrate is an inhibitor of carbonic anhydrase II enzymes as it is isoelectronic with carbonate^16^ and all enzymes were inhibited by the addition of NaNO_3_ at concentrations ranging from 50 to 3,000 mM. However, in terms of percentage of activity remaining compared with the control condition for each enzyme, M3 and M4 showed a significantly lower susceptibility to nitrate inhibition (Fig. 3c). That is, the concentration to inhibit activity by 50% (IC50) measured over range of substrate concentrations was significantly higher for M3 and M4 (Supplementary Table 2). Furthermore, the IC50 value increased with increased number of acidic residue substitutions on the carbonic anhydrase surface suggesting a strong influence of electrostatic interactions on the potency of nitrate inhibition.

Addition of the protein-denaturing ionic detergent SDS negatively affected the activities of all the enzymes; however, the esterase activity of M4 was least affected and its activity was significantly higher than the other carbonic anhydrases in the presence of SDS up to 0.2% w/v (Fig. 3d). The presence of GuHCl (250–5,000 mM) in the assay mixture did not discriminate between the WT and M3 as they were similarly inhibited but M4 was slightly more affected by the presence of the denaturant (Supplementary Fig. 9).

### Role of Na^+^ in stabilizing M4 structure

**Figure 4:**
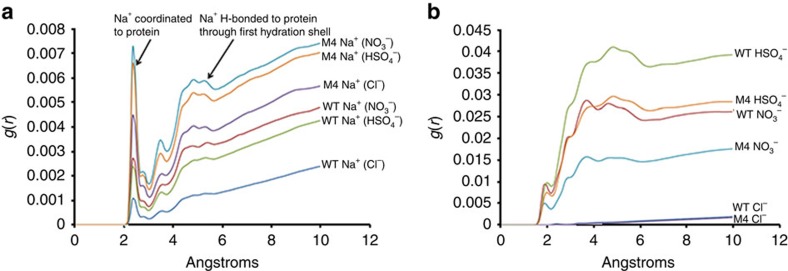
Radial distribution functions (*g*(*r*)) for cations and anions against whole enzymes. RDFs are shown from the MD simulations with WT and M4 across the three salt conditions for Na^+^ (**a**) and for the anions (**b**) calculated over 50,000 frames of the 500 ns production trajectory.

The molecular dynamics simulations revealed a complex variety of interactions between the cations, anions and enzymes. In general, Na^+^ cations interacted with more sites on M4 than with WT. The longest-lived Na^+^ residence times, that is, the time the cation was directly associated with the enzyme, occurred where chelating environments existed such as the presence of three or more coordinating oxygen atoms usually provided by a combination of Glu and Asp side chains and main chain carbonyl oxygen atoms. The radial distribution functions (RDFs) were calculated for the interaction of Na^+^ and enzyme surfaces from the NaCl, NaNO_3_ and NaHSO_4_ simulations. Four distinct association distances were apparent across the three salt simulations at 2.2, 2.7, 3.5 and ∼4.4–5.1 Å, with the first and last of these being the major peaks corresponding to directly coordinated Na^+^ and hydrogen-bonded fully hydrated Na^+^, respectively (Fig. 4). Comparison between the whole enzymes showed there was a clear increase in association of Na^+^ with M4 over WT in all salt conditions.

Analysis of the RDFs for the anions and proteins showed significantly lower populations of NO_3_^−^ and HSO_4_^−^ associating with M4 than with WT; however, this difference was not observed for Cl^−^, which showed almost no differences between the enzymes (Fig. 4). The shortest association peaks for NO_3_^−^ and HSO_4_^−^ occurred at ∼1.9 Å, whereas that for Cl^−^ was at 2.3 Å. There were four additional association peaks for NO_3_^−^ and HSO_4_^−^ indicating water-mediated interactions; however, they were slightly less well-defined for HSO_4_^−^. Cl^−^ showed two distinct peaks but at much lower levels of association compared with the polyoxoanions in their respective systems.

While the radius of gyration (Supplementary Fig. 10) for each protein over the course of the simulations indicated that there were no major perturbations to the secondary structure, as did visual inspection, the RMSD's for the M4 simulations in both NaNO_3_ and NaHSO_4_ began to gradually increase after ∼300 ns, the greatest being ∼3.8 Å for NaHSO_4_ at ∼570 ns. Interestingly, the same effect was not observed for the WT simulations. Overlaps of the covariance matrices for the first and second halves of the 500 ns production trajectory (Supplementary Table 3) suggest there may be some motions at much longer timescales that have not yet been fully sampled. The largest RMSD for the M4/NO_3_^−^ system is attributable to an outwards shift of the loop regions consisting of residues 198–204, whereas for the M4/HSO_4_^−^ system, it is primarily because of flexibility in the N-terminal region (approximately the first 10 residues) of the protein with a smaller outwards shift in the 198–204 loop compared with M4/NO_3_^−^. A more detailed analysis of the RDFs of Na^+^ with each mutated residue was performed and showed, for the majority of cases, very consistent association patterns both between the two halves of each simulation (Supplementary Figs 11–13). The greatest consistency was seen in the M4/HSO_4_^−^ system, which had also showed the greatest departure from the original structure in terms of backbone RMSD, indicating that there is sufficient sampling over the 500 ns to adequately capture the dominating cation/anion/protein associations. In the M4/Cl^−^ system there were discrepancies between the two halves of the simulation in the Na^+^ associations with D8, whereas in the M4/NO_3_^−^ system the main differences were in D8, D36, D62 and E238. The transient proximity of D62 and E238 to the flexible N-terminal region (which also contains D8) provides a highly changeable Na^+^ coordination environment, explaining the RDF differences at these residues. However the difference for D36, which is located in the centre of a loop on the opposite face to the active site and has no chelating partners, is due to randomly alternating ‘inward' and ‘outward' rotamers of this residue, which has it sharing a Na^+^ cation with D110 in the latter conformation only, and is not associated with any secondary structural movement. The longest-lived, consistent direct cation associations were at E169 across the Cl^−^, NO_3_^−^ and HSO_4_^−^ simulations for M4, where Na^+^ coordination was, interestingly, shared with the carbonyl oxygen atom of W5 despite the flexibility of the N-terminus. From the area under the curve for all the RDFs across all simulations, it is clear that the most common modes of association of the cations with the protein surface are actually mediated through the first hydration shells of the cations.

## Discussion

While it has been established that naturally occurring halophilic enzymes possess a combination of lower and smaller hydrophobic residue content, fewer lysine residues, reduced accessible solvent area and a negative surface charge compared with mesohalophiles^2^,^3^,^4^,^5^,^6^,^13^, here the sole basis of rational enzyme design was substitution of surface residues to produce enzymes with extreme halotolerance. Up to 18 amino acid substitutions were made to the enzyme surface of bCAII, but this did not significantly alter the secondary structure, catalytic activity or stability of the designed enzymes, compared with WT, in the presence of added salts. Surface residues that were not involved in significant interactions were targeted for substitution and this approach resulted in the loss of up to three Lys out of a total of 18 in the protein and a minor reduction in solvent-accessible surface area in the designed proteins. Traditionally, halophilic enzymes would be sourced from naturally occurring salt-tolerant organisms such as the alga *Dunaliella salina*; however, this approach does not always yield useful biocatalysts. Recombinant expression of halophilic enzymes in mesohalophilic organisms can be difficult because of their instability in low salt conditions^17^ and for a carbonic anhydrase from *D. salina*, the component subunits lacked haloterance and CO_2_ hydration activity^18^. Moreover, other rational design approaches that target surface residues with ‘supercharging' algorithms for increasing protein stability^19^ have had limited application to catalytic proteins and, to the best of our knowledge, supercharged proteins have not been reported to be halotolerant. In light of recent crystallographic findings showing that increased numbers of acidic surface residues disrupt ordered pentagonal water networks^20^, it is perhaps unsurprising that the mutants' thermostabilities were reduced in low-salt conditions compared with WT. Our approach to rational design for halotolerance, which involves selective conversion of surface residues to acidic residues and distributing residue substitutions across the surface, has meant the resulting enzymes were not as destabilized as may be expected by altered electrostatics^21^ or because of the large number of residue changes^22^.

Undertaking the residue substitutions in the WT enzyme in stages and investigating the separate and combined substitutions allowed the intermediate enzymes with fewer substitutions to be assessed. Generally, the salt tolerance of enzymes increased with increasing number of substitutions, as measured by catalytic activity and thermostability, with M4 showing the greatest halotolerance. In low-salt conditions (<1 M), M3 and M4 were less stable than WT as demonstrated by both lower unfolding temperatures and catalytic activities. The phenomenon of reduced stability in halophilic proteins in low-salt conditions has been attributed to either a reduction in the hydrophobic effect^4^ or repulsive electrostatic interactions^19^. A significant change in the hydrophobic effect is unlikely for M3 and M4 compared with WT as the buried hydrophobic core residues of the proteins were unaltered in the designed versions.

**Figure 5:**
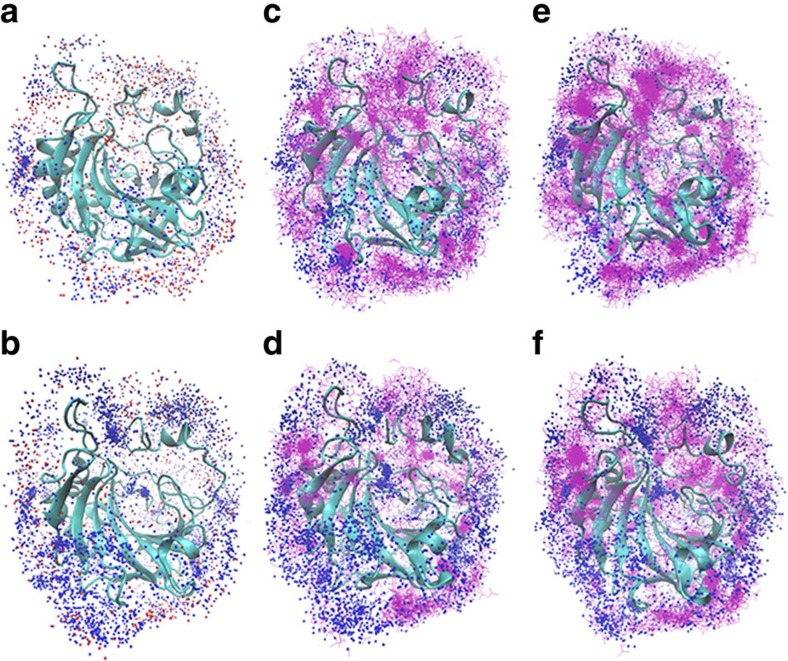
Overlays showing the distribution of cations and anions within 3 Å of the enzyme surface. The overlays incorporate anions and cations from 500 frames spanning the 500 ns MD production trajectories. (**a**,**b**) NaCl with WT and M4, respectively; (**c**,**d**) NaNO_3_ with WT and M4, respectively; and (**e**,**f**) NaHSO_4_ with WT and M4, respectively. Na^+^ cations are depicted as dark blue spheres, HSO_4_^−^ and NO_3_^−^ anions are depicted as purple lines and Cl^−^ anions are depicted as orange spheres.

Significantly, M3 and M4 were stabilized by the addition of salts to the media for thermal unfolding analysis (Fig. 1) and the extent of stabilization followed the Hofmeister series, that is, anions of high charge density, such as sulfate, were more effective in stabilizing M1–M4 followed by chloride then nitrate, with all salts sharing sodium as the cation. Indeed, the thermostability regained by M4 in high-Na_2_SO_4_ and -NaCl solutions (Fig. 1a,b) suggests WT was transformed into an obligate halophile through engineering of its surface residues. The increase in the thermostabilities of M1–M4 in the presence of salts was mirrored by the increased esterase activity of the enzymes in the presence of sodium salts of sulfate and chloride relative to WT (Fig. 3). Indeed, the activities of M3 and M4 in 1,500 mM sulfate were significantly enhanced relative to WT and well in excess of the ‘salting out' effect of substrate observed previously by Steiner and Linskog^23^. In that study, Na_2_SO_4_ at concentrations of up to 1 M caused a marked increase in 4-NPA hydrolysis activity by human CAII and this was attributed to a salting out effect of the dissolved substrate with little or no salt effect on the enzyme active site residues. In that study, the activity increase reached a plateau at pH values above 8 in the presence of 1,000 mM Na_2_SO_4_ and this was approximately four-fold higher than the rate in the absence of salts, which was consistent with the WT increase in Fig. 3a. Sodium nitrate, together with most monovalent anions, inhibits carbonic anhydrase through binding to the active site at a position close to the metal ion and displaces the metal-coordinated hydroxide ion involved in the catalytic reaction^24^. However, despite the active site residues remaining unaltered in M1, M3 and M4, sodium nitrate was significantly less inhibitory of the esterase reaction of these enzymes compared with WT. This could be due to the decreased apparent local concentration of nitrate around the enzyme, as observed in the molecular dynamics (MD) simulations (Fig. 5). We also note that an increased concentration of Na^+^ ions around the active site in the MD simulations prevents nitrate from otherwise occupying the area (Fig. 5c,d).

The engineered halotolerant enzymes were also more resistant to the destabilizing effects of selected ILs and denaturants such as SDS that cause unfolding of proteins through the disruption of secondary structural elements (α-helices and β-sheets). SDS denatures proteins through disruption of tertiary structure via unfolding and chain expansion, depending on the micelle concentration^25^. SDS was less effective in disrupting the structure and catalytic activity of M4 presumably because of the repellent charge effects on the surface of the engineered enzyme that reduced access of negatively charged micelles to the hydrophobic core. In addition, the number of exposed hydrophobic residues is reduced in M4, hence providing fewer points of access for the SDS hydrophobic tail. However, for denaturants such as urea (neutral) and GuHCl (positively charged), the engineered enzymes were similarly susceptible as the WT enzyme.

The salt ion interactions were explored with the WT and M4 enzymes using molecular dynamics. Highly specific interactions with particular groups of residues were noted over the course of the simulations. As expected, an increase in surface acidic residues in M4 had the subsequent effect of increasing the population of Na^+^ at the surface and also in secondary shells connected to the surface by waters of hydration. This had a shielding effect whereby Na^+^ occupied significant regions of surface-space for prolonged periods and prevented the approach of anions (Fig. 5). This effect is clear from the RDFs for HSO_4_^−^ and NO_3_^−^, but was not observed for Cl^−^. This could be due to the significantly smaller hydrodynamic radius of Cl^−^ allowing a more efficient packing of hydrated Na^+^Cl^−^ layers around the surface, somewhat negating the exclusion effect of Na^+^. A comparison of the Cl^−^ distributions around WT and M4 shows that Cl^−^ can approach the surface in a much more diffuse area for WT, whereas M4 shows much more well-defined pockets where Cl^−^ can reside with much longer residence times (Fig. 5).

RDFs for the individual substituted residues introduced into M4 and not present in WT showed a clear preference of some for Na^+^ over others. This was largely dictated by coordination number, with chelating environments strongly favouring long residence times. Acidic residues isolated from chelating partners were still able to attract Na^+^ cations; however, the interactions were highly transient, usually with a single water being displaced from the first Na^+^ hydration sphere and then rapidly replaced from the bulk solvent. The most significant observation from the RDFs is that the dominant associations of the cations with the new acidic residues were actually those mediated through the hydration shells of the cations. These were complex and dynamic associations whose prevalence suggests that it is actually the hydrated cations, specifically, their ordered hydration shells, that act to re-establish a stabilizing surface-water network that was disrupted when the naturally evolved surface was altered. Future halotolerance design strategies should take this into account; however, it would be necessary to carry out additional calculations using a more sophisticated water model and timesteps of finer resolution to further investigate the nature of the hydrated cations' influence on the broader water network.

The many biophysical and compositional features of halophilic enzymes that distinguish them from mesohalophiles have been drawn from comparative genome, proteome and crystal structure analyses. In contrast to suggestions that large differences in accessible solvent area, non-polar surface area or absence of large hydrophobic amino acids are the important features that allow halophilic enzymes to function optimally in high-salt environments, we have found that increasing the number and distribution of surface acidic groups alone is sufficient to impart this characteristic. The design principles demonstrated here could be readily applied to other mesohalophilic enzymes. Furthermore, we find that increased frequency and longevity of binding of cations to acidic residues on the halotolerant enzyme surface has the dual effect of stabilization of the surface structure and increased repulsion of anions.

## Methods

### Rational design of carbonic anhydrase for haloterance

**Figure 6:**
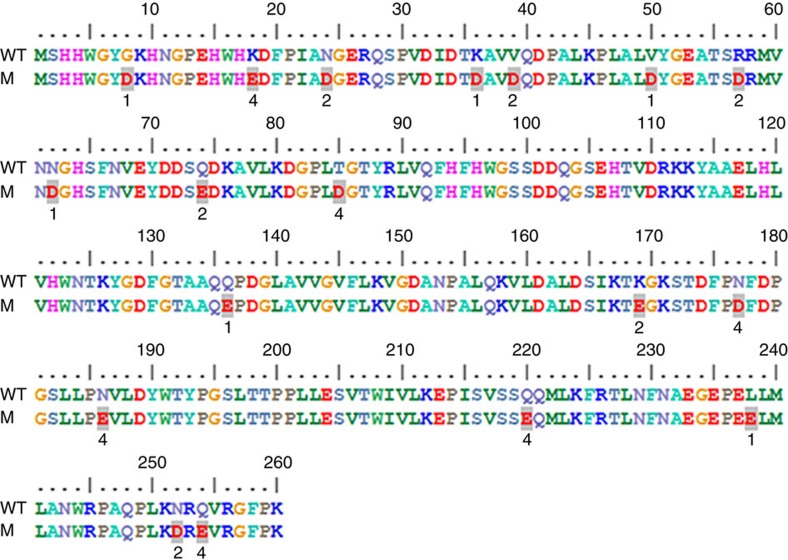
Alignment of the WT amino acid sequence with the rationally designed, halotolerant enzymes (M). The shaded residues were altered in the designed sequences versus WT and the sequence where each substitution featured are given in the bolded number below, that is, the change is uniquely present in either M1, M2 or M4.

**Figure 7:**
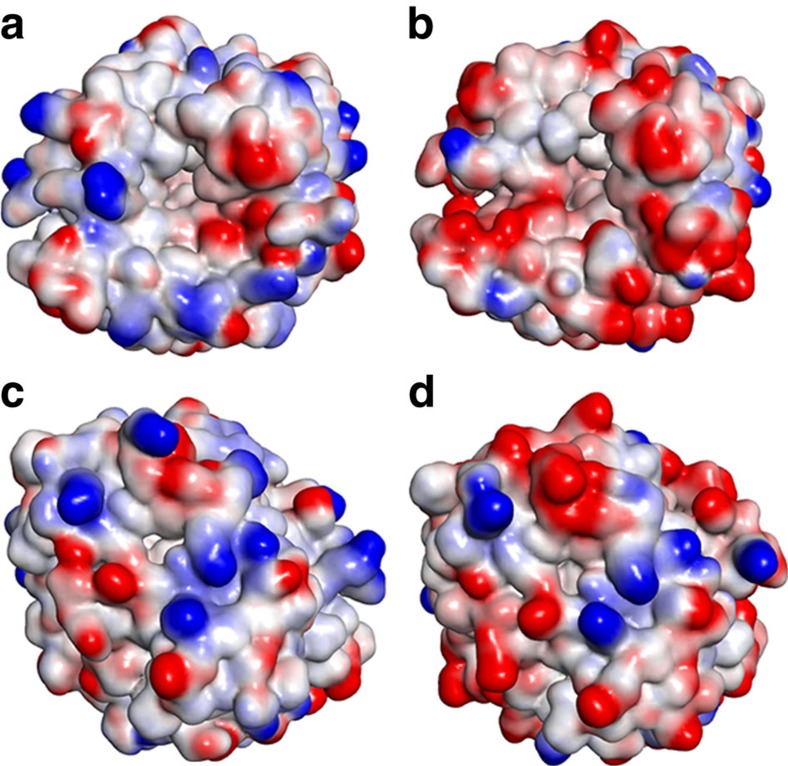
Distribution of surface charge in WT and M4. Positive charges (blue) and negative charges (red) are projected onto a solvent-accessible surface (1.4 Å probe radius) of WT (**a**,**c**) and designed M4 (**b**,**d**) carbonic anhydrase II using an interpolated charge algorithm as implemented in Discovery Studio 3.5. The top images, looking directly into the active site pocket, are rotated 180° around a vertical axis compared with the bottom images.

The amino acid sequence of bovine carbonic anhydrase II (bCAII; NP_848667.1) was subjected to BLASTp analysis and the 50 best matching sequences (Supplementary Data 1) were aligned using ClustalW. In most cases, residue positions in bCAII that were most variable with respect to amino acid in the sequence alignment with other carbonic anhydrases were selected as candidates for mutation as they were considered to be the most tolerant of variation (Supplementary Fig. 14). Preference of site for mutation was given to residues that were on the surface of the protein as determined by examination of the bCAII crystal structure (PDB: 1V9E) and not already involved in significant interactions such as salt bridges or hydrophobic interactions with adjacent residues to minimize potential negative impacts on stability. The decision of whether to mutate an existing residue, most usually neutral, to an acidic residue (Asp or Glu), was based largely on steric bulk of the side chain being replaced with some consideration being given to whether a new salt bridge could be generated or an existing one maintained. A total of 18 residues in total were selected for mutation; however, as we were unable to predict how many changes it may take to confer halotolerance to a salt-susceptible enzyme or how many residue changes would be tolerated by the enzyme structure before it became unstable or affected folding, the mutations were introduced in stages. Firstly, two independent sets of six amino acid substitutions each were introduced into WT (M1 and M2, Table 3) with a view to maximize the spatial arrangement across the enzyme's surface within each group and enable the assessment of stability and activity of each set. Following this, a combination set of these 12 mutations was also generated and assessed (M3). A final set of an additional six changes were added into the 12 changes already present in M3 to generate enzyme M4. The locations of the residue changes in the designed enzymes are summarized in the sequence alignment (Fig. 6) and the extent of change in surface charge for the M4 enzyme compared with WT is shown in Fig. 7.

### Recombinant expression and purification

Native bCAII was purchased from Sigma Aldrich and was used as supplied or purified via IMAC affinity chromatography as described below. Recombinant bCAII (NM_178572.2), herein described as ‘wild type', and the designed enzymes M1–M4 (Table 3) were synthesized in codon-optimized versions (Geneart, Life Technologies) and ligated in frame into pET28 using the *XhoI* and *NcoI* restrictions sites. The in-frame insertion resulted in the introduction of two additional non-native amino acids (MG) at the N-terminus and did not incorporate the histidine-tag. Plasmids with the correct insert were transformed into *E. coli* BL21(DE3)* strain, positive colonies were selected and cultures prepared for expression in autoinduction media. The DNA sequence of the plasmid insert coding for M4 was reconfirmed and the expressed M4 protein purified from two separate *E. coli* cultures was excised from SDS–polyacrylamide gel electrophoresis, trypsin digested and the peptide fragments analysed by Agilent electrospray LC-MS. Peptides were matched to sequences in the NCBI non-redundant protein database and the predicted protein M4 using Spectrum Mill software. Peptides for the expressed M4 sequence completely matched the predicted sequence. At the completion of the incubation period, the cells were pelleted, lysed in buffer containing 0.5 mM ZnCl_2_ (WT only) and bound to a Ni-NTA column for purification by fast protein liquid chromatography. The WT and engineered enzymes were eluted with an imidazole gradient and the fractions containing carbonic anhydrase were dialysed against 50 mM Tris-HCl at pH 7.5 containing 10 μM ZnCl_2_ (except M4). For M4, dialysis against ZnCl_2_ was performed at 0.5 mol mol^−1^ enzyme. All characterization reported herein was performed on the Zn-supplemented enzymes.

### Crystallization of mutants

Thermal denaturation studies were used to select a formulation buffer (50 mM piperazine pH 5.5, 50 mM NaCl) for crystallization. M1–M4 were dialysed into this buffer then screened at approximately 20 mg ml^−1^ against a sparse matrix screen and an ammonium sulfate/Tris buffer screen based on the known crystallization conditions for bovine carbonic anhydrase II. Diluting the protein to 10 mg ml^−1^ and setting up against the JCSG+ sparse matrix screen at 8 °C gave crystals of M1 and M3, these were used to seed further screening experiments with the M2 and M4 protein also at 8 °C. Eventually, after several rounds of seeding or cross-seeding, where the droplets are seeded with a seedstock produced from crystals of another mutant, crystals were obtained for all four variants. All crystallization experiments were set up in droplets consisting of 150 nl protein solution, 140 nl reservoir solution, 10 nl seedstock against 50 μl of reservoir in SD-2 sitting drop plates (Molecular Dimensions, UK) and employing Phoenix (ARI, CA) and Mosquito (TTPLabtech, UK) automation to set up the droplets.

The crystallization conditions for the crystals used for data collection were as follows: M1: 30% w/v PEG 1,500; M2: 5% w/v PEG 1,000, 30% w/v PEG 600, 100 mM sodium MES pH 6, 10% glycerol; M3: 25% w/v PEG MME 2k, 200 mM calcium acetate, 50 mM Tris-HCl pH 8; and M4: 40% w/v PEG 600, 100 mM sodium citrate pH 5.5.

### X-ray crystal structures of mutants

X-ray data were collected at 100 K at the MX-1 beamline at the Australian Synchrotron (0.95370 Å wavelength). The crystals from each mutant were cryo-cooled in the nitrogen stream just using reservoir as the cryo-protectant. In each case, 360 1° oscillations were obtained for a total of 360° of data. The data were indexed using XDS^26^, and scaled and truncated using SCALA/TRUNCATE^27^. Phaser^28^ was used for molecular replacement, using PDB 3ML2 as the starting model. Models were refined using Refmac^29^ after rebuilding by hand using Coot^30^. Ramachandran statistics were as follows: M1: 92.9% favoured, 6.7% allowed and 0.4% outliers; M2: 93.9% favoured, 5.6% allowed and 0.4% outliers; M3: 93.2% favoured, 6.8% allowed and 0.0% outliers; and M4: 91.9% favoured, 7.1% allowed and 1.0% outliers.

### Enzyme unfolding analysis

High throughput screening for thermostability of WT and designed enzymes was conducted in the presence of various buffers, salts, denaturing agents and IL solutions using DSF^31^. The assay was performed in 96- or 384-well PCR plates in a final volume of 20 μl containing enzyme (∼0.3 μg), SYPRO dye (0.3 μl of a 1:10 dilution of the dye in water) and 19.4 μl of the test solution. The plates were heated at 1 °C min^−1^ between 20 and 100 °C in a BioRad CFX96 (or CFX384) PCR machine and in each batch performance controls were included such as commercial bovine carbonic anhydrase (Sigma) in 50 mM Tris-HCl pH 7.5, lysozyme and dye controls. Each enzyme and condition was measured in triplicate and the values were averaged.

The conditions tested included a buffer screen (50 mM) over the pH range 7–11 in 50 mM Tris-HCl. The following salts were added at the stated concentrations: 1,000–3,000 mM NaCl; 250–2,000 mM Na_2_SO_4_; 500–3,000 mM NaNO_3_; and each of guanidinium hydrochloride (GuHCl), urea, ethyl ammonium formate, ethyl ammonium nitrate, ethanol ammonium formate (EtOH-AF) and 1,3-dimethylimidazolium dimethylphosphate (DmimDMP] at 50–2,000 mM.

For each sample run, the unfolding curve shape, starting points and end points were examined and the temperature of hydrophobicity (*T*_h_) was estimated as a function of pH, salt concentration and the buffering or additive chemical. The value *T*_h_ has been previously shown to be highly correlated to the absolute melt temperature, *T*_m_ (ref. 32). Prior experience with the technique^31^ indicates the inherent variability in the experimental system is ±1 °C, such that *T*_h_ changes of >1 °C are significant.

### Structure assessment by circular dichroism spectroscopy

Far UV CD spectra were obtained at 1 nm intervals between 190 and 250 nm on a Chirascan spectrometer (Applied Photophysics) using a 0.2 mm path length quartz cell (20/O/Q/0.2, Starna) at 25 °C. Buffer for the analysis was 10 mM Tris-HCl pH 8. Spectra of a blank solution, which contained all assay components except protein, were recorded for each additive concentration. Protein concentration was adjusted to achieve optimal absorbance (1.3–1.6 AU) between 209 and 220 nm. Blank and sample spectra were each measured in triplicate; mean absorbance for blank spectra were subtracted from mean sample absorbance to derive final CD spectra. α-Helix and β-sheet content of enzymes in solution in the presence and absence of GuHCl (250–5,000 mM) or SDS (0.01–0.5% w/v) were estimated using the program K2D3 (ref. 33) using the obtained CD spectra values. Denaturant was added to enzymes before CD analysis or pre-incubated with enzymes for 1 or 24 h.

### Catalytic activity and effect of salts and inhibitors

Carbonic anhydrase esterase activity was measured by the spectrophotometric method of Pocker and Stone^34^ with 4-nitrophenyl acetate (4-NPA) as substrate with the following modifications: buffer was 50 mM HEPES pH 8 containing 50 mM Na_2_SO_4_ (ionic strength (*I*)=0.2 M). Production of 4-nitrophenol was monitored at 405 nm over 10 min at 25 °C using a Molecular Devices SpectraMax Plus spectrophotometric plate reader and the concentration of 4-nitrophenol product determined by calibration curves prepared over a 0.01–0.25 mM concentration range. Salts, detergents, denaturants and ILs that were assessed for their effect on activity, were fully mixed into the assay buffer before the addition of substrate to initiate the reaction. Na_2_SO_4_ (1 M final concentration) had no effect on the absorbance measurement for 4-nitrophenol (0.1 mM) when added to the assay buffer. Appropriate control samples lacking enzyme or substrate were included and the background hydrolysis rate of 4-NPA did not exceed 6% of the catalysed rate for any assay mixture tested. The uncatalysed hydrolysis rates were subtracted from the corresponding enzyme catalysed rates. In addition, to determine whether there was any influence of assay pH on esterase activities between WT and engineered enzymes, assays were measured by production of 4-nitrophenol measured at its isosbestic point (348 nm) and lambda max (405 nm) over the pH range 6.8–8 with 4-NPA (0.5 mM) at 28 °C, and product formation was monitored over 10 min.

Sodium nitrate inhibition of WT and designed carbonic anhydrases was examined over the concentration range 50–3,000 mM at eight substrate 4-NPA concentrations between 90 and 2,000 mM. The inhibitor concentration required to reduce activity by 50% (IC50) was determined for each of the enzymes at each substrate concentration.

To determine the effect of urea on 4-NPA activity of bCAII and engineered enzymes, the denaturant was added to assay mixtures at a final concentration of between 500 and 4,000 mM with a final substrate concentration of 1 mM and otherwise conducted under the standard assay conditions described above. Similarly, the effect of Na_2_SO_4_ over the range 250–1,850 mM, NaCl between 750–3,000 mM and GuHCl between 250–3,000 mM were assessed by enzyme assay.

### *In silico* modelling and calculations

MD simulations were carried out on using the WT and M4 crystal structures as starting geometries to investigate the differences in salt–protein interactions and to attempt to rationalize the differences in thermostability under different salinity conditions. Simulations were performed using AMBER14 (ref. 35) and employed the ff99SB-ildn* force field^36^,^37^ for non-coordinating protein residues and the zinc AMBER force field^38^ for the Zn^2+^ coordination environment, which included a coordinated hydroxide group. Na^+^ and Cl^−^ parameters were taken from Joung and Cheatham^39^. The NO_3_^−^ and HSO_4_^−^ structures were optimized using DMol3 with all fine-grained defaults as implemented in Accelrys Materials Studio 7.0 (ref. 40) employing the PBE functional^41^ and the DNP basis set (double numerical quality with polarization functions comparable to the 6–31G** basis set). The anions were parameterized for MD employing BCC charges using the antechamber code as implemented in AmberTools15 (ref. 35) and treated with the General AMBER force field for simulation. For the NaCl simulations, the WT and M4 structures were neutralized and had further Na+ and Cl- ions added (using the addions2 command in xLEaP) to provide a final count of 130 additional NaCl. For the NaNO_3_ or NaHSO_4_ simulations, the WT and M4 enzymes were first neutralized with Na^+^ cations, after which single units of NaNO_3_ or NaHSO_4_ were created and then added in excess to the systems using the solvate shell command xLEaP. The ions were then pared back to provide 130 NaNO_3_ or NaHSO_4_ above the neutralizing Na^+^ cations using an in-house python script. All systems were finally solvated in octahedral TIP3P water boxes, the sizes of which were adjusted to provide approximately equal total concentrations for the systems (final system parameters are provided in Supplementary Table 3). Although not expected to be the dominant species at our experimental pH levels, NaHSO_4_ was used instead of Na_2_SO_4_ due to the latter forming structured clusters in early simulations. All simulations were performed at constant pressure using the Berendsen barostat with isotropic position scaling (NTP=1) and a pressure relaxation time of 2 ps. The Langevin thermostat was used for temperature regulation. Long range electrostatic interactions were treated with the particle mesh Ewald method beyond 12 Å. Each system was minimized to relax high-energy contacts while restraining the protein backbone. Simulations were carried out at 298 K for 600 ns employing a 2 fs timestep with SHAKE constraints and a non-bonded distance cutoff of 12.0 Å. Coordinates were saved at 10 ps intervals to give final trajectories consisting of 60,000 frames. Inspection of the RMSD plots for the protein backbone atoms and the radius of gyration for each system indicated that the simulations had typically stabilized by 50–100 ns, hence the first 100 ns was discarded and all analysis was carried out on the final 500 ns. Enzyme images were produced and physical parameters were calculated using either Discovery Studio 3.5 (ref. 42), VMD^43^ or cpptraj within AmberTools15.

## Additional information

**Accession codes:** Coordinates and structure factors for M1, M2, M3 and M4 have been deposited in the RCSB Protein Data Bank under accession codes 4CNR, 4CNV, 4CNW and 4CNX, respectively.

**How to cite this article:** Warden, A. C. *et al*. Rational engineering of a mesohalophilic carbonic anhydrase to an extreme halotolerant biocatalyst. *Nat. Commun.* 6:10278 doi: 10.1038/ncomms10278 (2015).

## Supplementary Material

Supplementary Information Supplementary Figures 1-14 and Supplementary Tables 1-3

Supplementary Data 1 Top 50 BLAST hits against the nr protein database for Bovine Carbonic Anhydrase II - Rational engineering of a mesohalophilic carbonic anhydrase to an extreme halotolerant biocatalyst

## Figures and Tables

**Table 1:** Protein crystallization data obtained for WT and designed enzymes (M1–M4).

	**WT**	**M1**	**M2**	**M3**	**M4**
*Data collection*					
Space group	P2_1_	P2_1_	P2_1_	P2_1_	P2_1_
Cell dimensions					
*a*, *b*, *c* (Å)	59.3, 79.4, 61.5	45.3, 141.1, 77.2	41.8, 69.3, 44.6	42.2, 134.3, 46.8	41.6, 67.9, 43.1
α, β, γ (°)	90.0, 106.98, 90.0	90.0, 90.07, 90.0	90.0, 107.76, 90.0	90.0, 104.04, 90.0	90.0, 103.07, 90.0
Resolution (Å)*	1.88 (1.98–1.88)	2.29 (2.41–2.29)	1.62 (1.70–1.62)	2.03 (2.14–2.03)	1.23 (1.30–1.23)
*R*_merge_	0.087 (0.378)	0.117 (0.391)	0.107 (0.637)	0.071 (0.181)	0.043 (0.323)
*I*/*σI*	19.5 (4.4)	14.2 (5.1)	16.3 (3.2)	22.9 (10.8)	28.7 (6.2)
Completeness (%)	93.8 (68.3)	98.2 (98.0)	99.9 (99.5)	92.4 (96.1)	98.6 (95.2)
Redundancy	7.4 (5.5)	75. (7.7)	7.4 (7.3)	7.5 (7.7)	7.3 (7.2)
					
*Refinement*					
Resolution (Å)	40.0–1.33	44.7–2.38	34.7–1.62	38.0–2.03	35.8–1.23
No. reflections	38,944	19,687	29,409	28,407	62,737
*R*_work_/*R*_free_	15.3/19.7	15.5/19.7	14.3/18.2	21.5/25.9	11.1/14.7
No. atoms	4,643	3,591	2,365	4,401	2,641
Protein	4,187	3,400	2,103	2,067	2,228
Ligand/ion	2	2	1	3	1
Water	386	163	249	281	405
*B*-factors	17.4	22.4	12.1	14.5	12.4
Protein	16.75	22.3	11.1	14.4	10.3
Ligand/ion	11.1	27.3	8.1	14.2	6.7
Water	23.6	21.6	20.1	16.1	23.6
R.m.s deviations					
Bond lengths (Å)	0.016	0.006	0.023	0.009	0.021
Bond angles (°)	1.766	1.249	2.104	1.269	2.069

WT, wild type.

^*^A single crystal was used for each structure.

**Table 2:** Catalytic activities for WT and designed enzymes (M1–M4).

**CAII enzyme**	Esterase activity* **(μmol min**^−1^** nmol**^−1^**)**	**CO**_**2**_ **hydration (M**^−1^ **s**^−1^**) × 10**^8^†
WT	2.12	1.198
M1	1.30	1.037
M2	1.16	1.034
M3	0.71	0.928
M4	0.71	0.957

WT, wild type.

^*^Substrate is 4-nitrophenyl acetate in 50 mM Tris-HCl pH 8.5 (*I*=0.2 M).

^†^From Phan *et al*.^44^.

**Table 3:** Amino acid substitutions generated for rationally designed carbonic anhydrase II enzymes examined in this study.

**Protein**	Amino acid substitutions compared with WT as expressed*
M1	G8D, K36D, V50D, N62D, Q136E and L238E
M2	N24D, V39D, R57D, Q74E, K169E and N252D
M3	G8D, N24D, K36D, V39D, V50D, R57D, N62D, Q74E, Q136E, K169E, L238E and N252D
M4	G8D, K18E, N24D, K36D, V39D, V50D, R57D, N62D, Q74E, T85D, Q136E, K169E, N177D, N186E, Q220E, L238E, N252D and Q254E

WT, wild type.

^*^Cloning of WT and designed enzymes into the expression vector pET28 resulted in two additional residues at the N-terminus of the protein sequence (MG) relative to the native sequence, but the original numbering has been conserved here.
