# Supplementary material for: Rational engineering of a mesohalophilic carbonic anhydrase to an extreme halotolerant biocatalyst
Source: Nat Commun. 2015 Dec 21;6:10278. doi: 10.1038/ncomms10278 (PMC4703901; doi:10.1038/ncomms10278)
Supplement: Supplementary Information — Supplementary Figures 1-14 and Supplementary Tables 1-3 [file ncomms10278-s1.pdf]

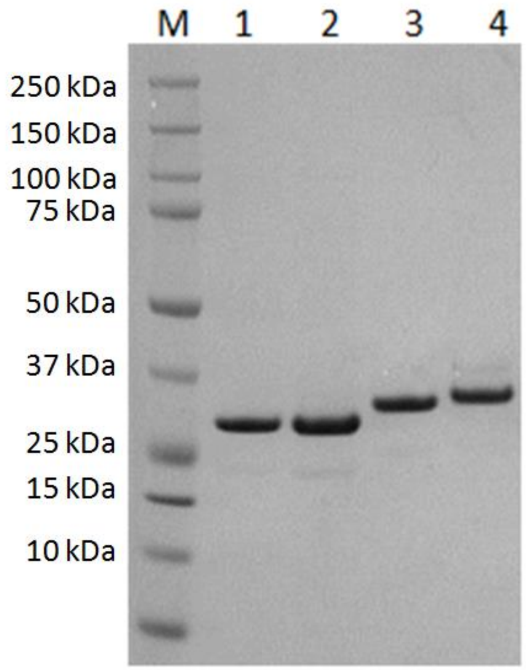

Supplementary Figure-1. **SDS PAGE analysis of purified designed carbonic anhydrase enzymes.** M1-M4 shown in lanes 1-4, respectively, with molecular weight markers (M).

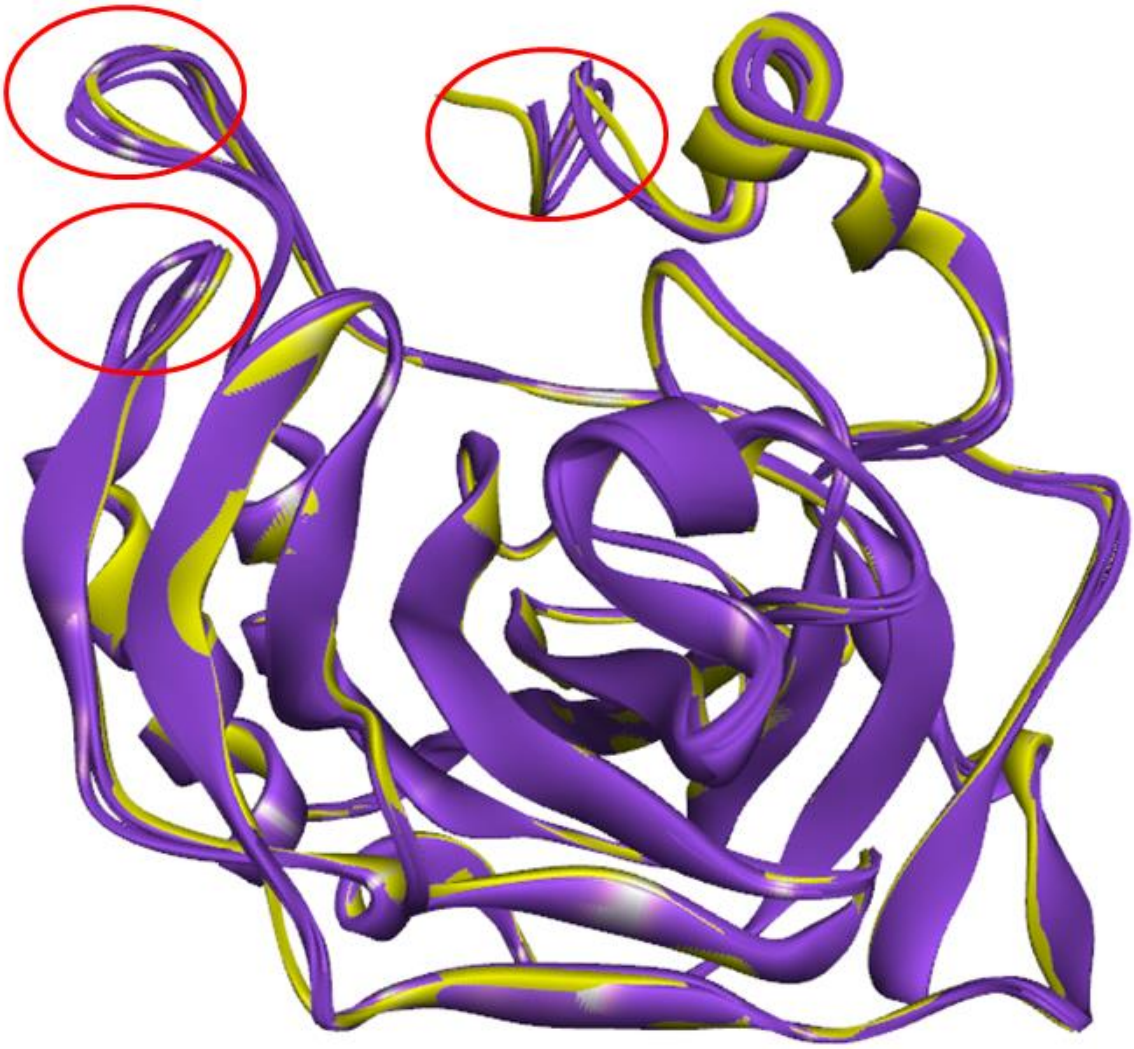

Supplementary Figure-2. **Overlay of the crystal structures of the four mutants (purple) and the wild type (yellow)**. M1-M4 are represented in purple and the WT in yellow. The loop regions where small amounts of variability can be seen are indicated by red circles.

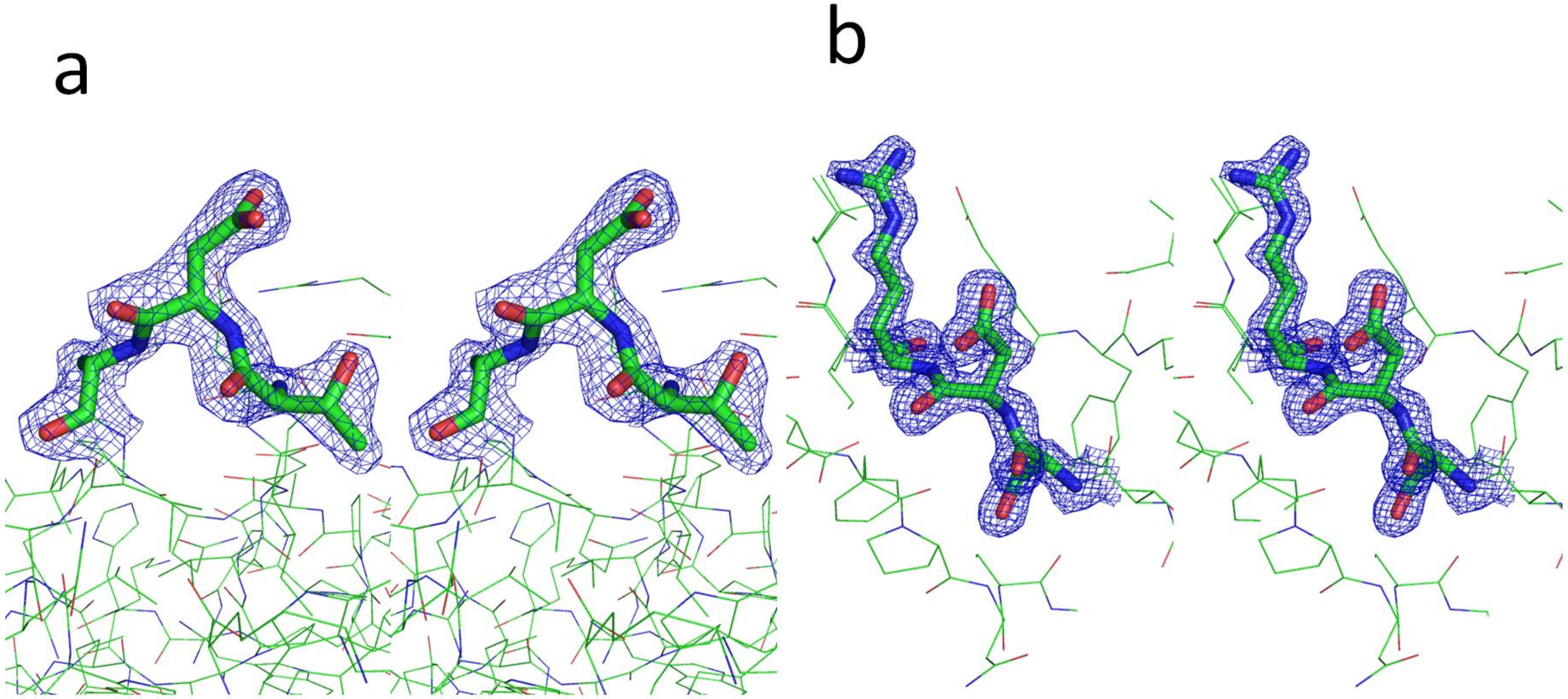

Supplementary Figure-3. Electron density maps of selected mutated residues in M1-M4. Three residues are shown in each panel: n-1, n and n+1 around the mutation. 2mFo-DFc electron density maps are shown at the 1.5 sigma level. (a) n = D36 in M1 and (b) n = D57 in M2.

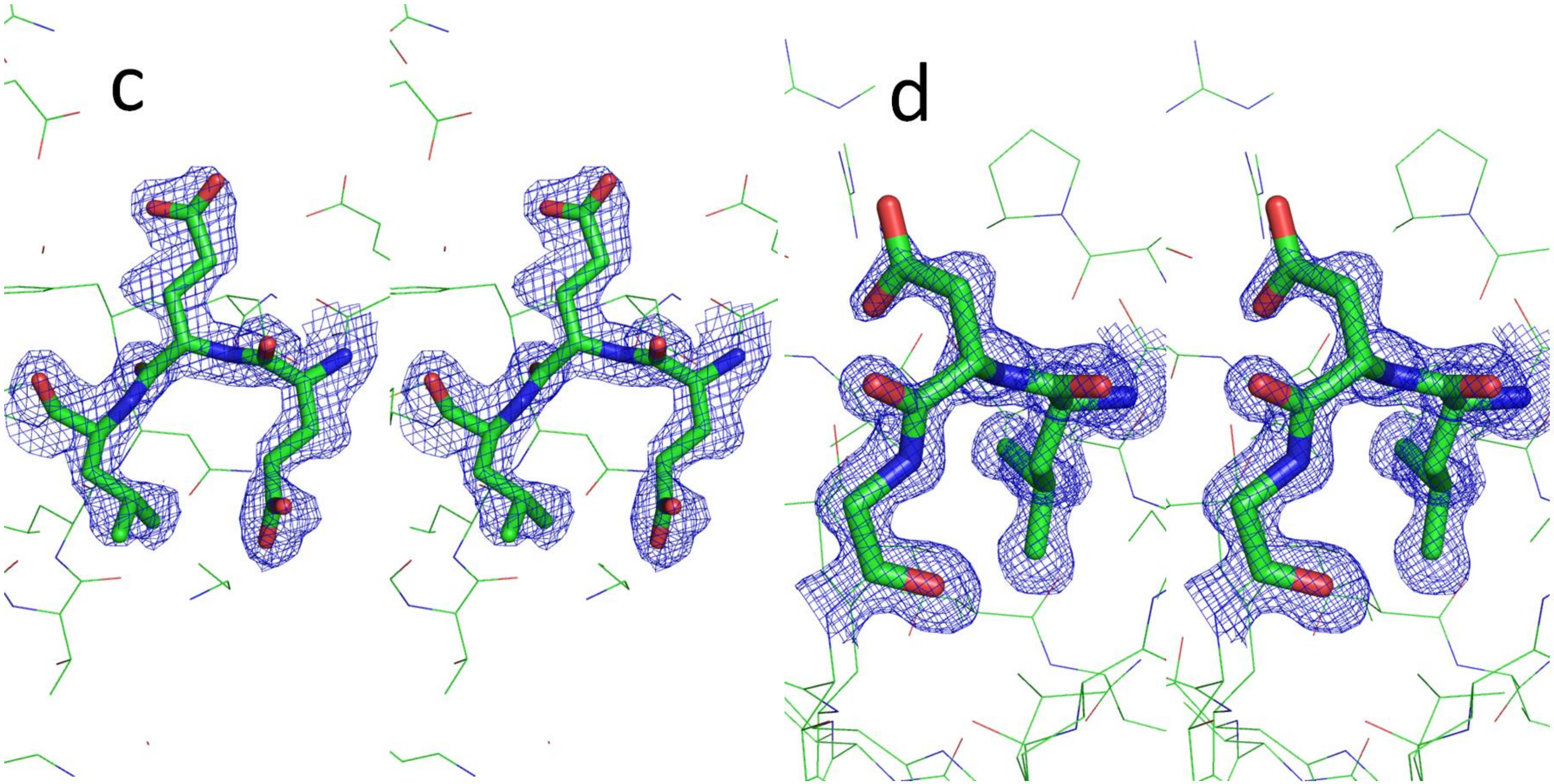

Supplementary Figure-3 (continued). Electron density maps of selected mutated residues in M1-M4. Three residues are shown in each panel: n-1, n and n+1 around the mutation. 2mFo-DFc electron density maps are shown at the 1.5 sigma level. (c) n = E238 in M3 and (d) n = D86 in M4.

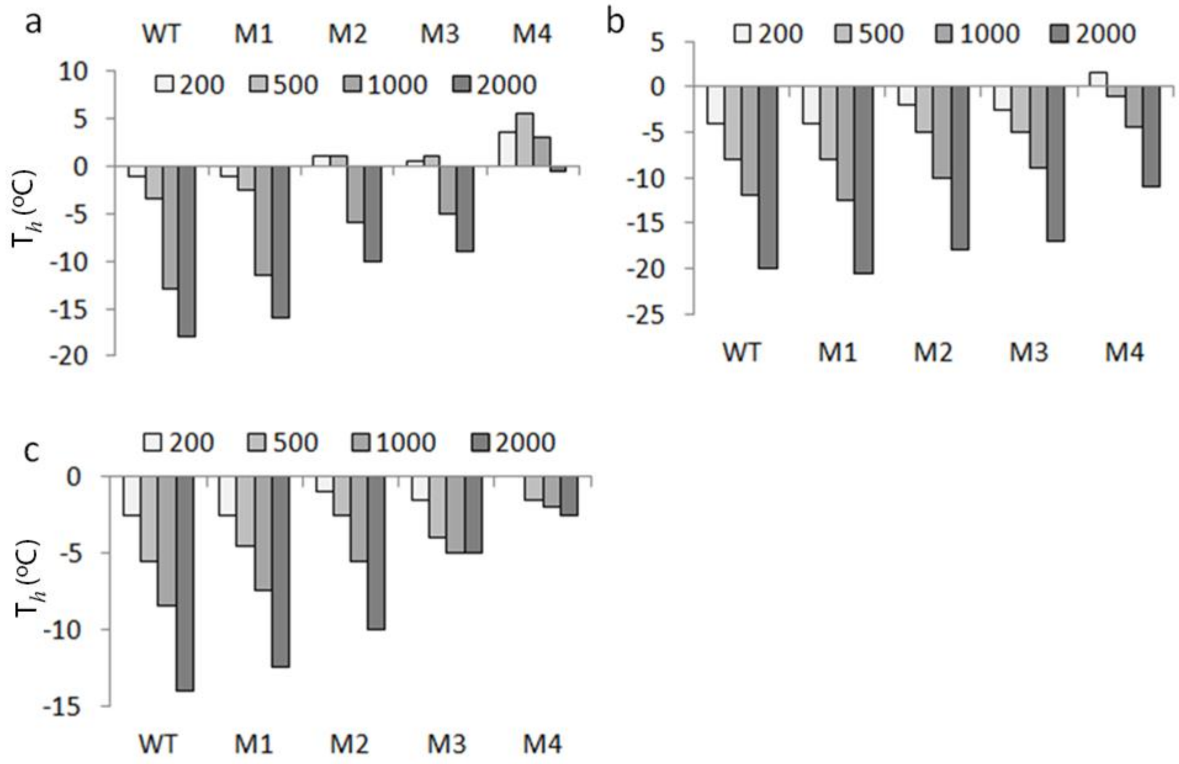

Supplementary Figure-4. **Thermal unfolding analysis for WT and M1-M4 carbonic anhydrases**. Experiments were performed in the presence of increasing millimolar concentrations of ionic liquids (a) Ethyl-AF (b) Ethyl-NO_3 and (c) DMI-DMP. For each enzyme, thermostability is indicated by the difference in thermal unfolding value (T_h) obtained by DSF in the presence of ionic liquid and 50 mM Tris-HCl pH 8.5 versus buffer only.

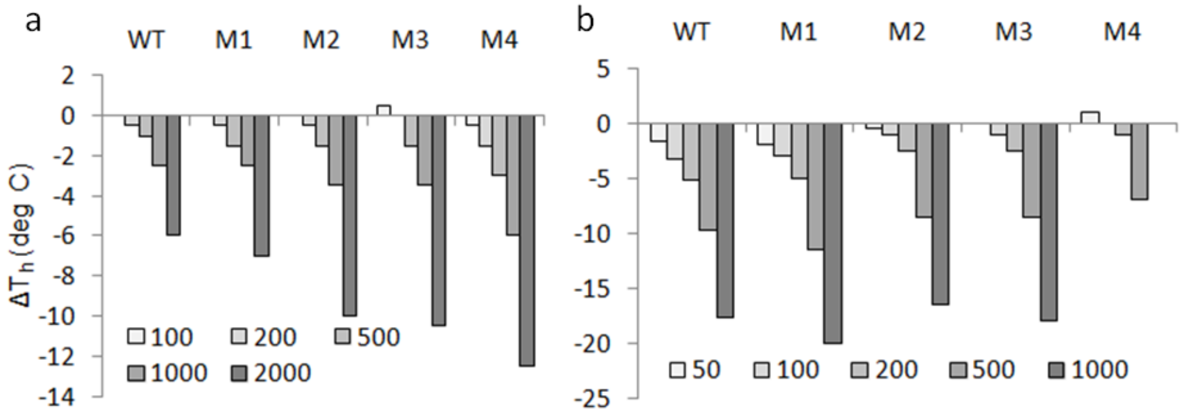

Supplementary Figure-5. **Thermal unfolding analysis of WT and M1-M4 carbonic anhydrases**. Experiments were performed in the presence of increasing millimolar concentrations of denaturants (a) urea and (b) guanidinium hydrochloride. For each enzyme, thermostability is indicated by the difference in thermal unfolding value (T_h) obtained by DSF in the presence of denaturant and 50 mM Tris-HCl pH 8.5 versus buffer only.

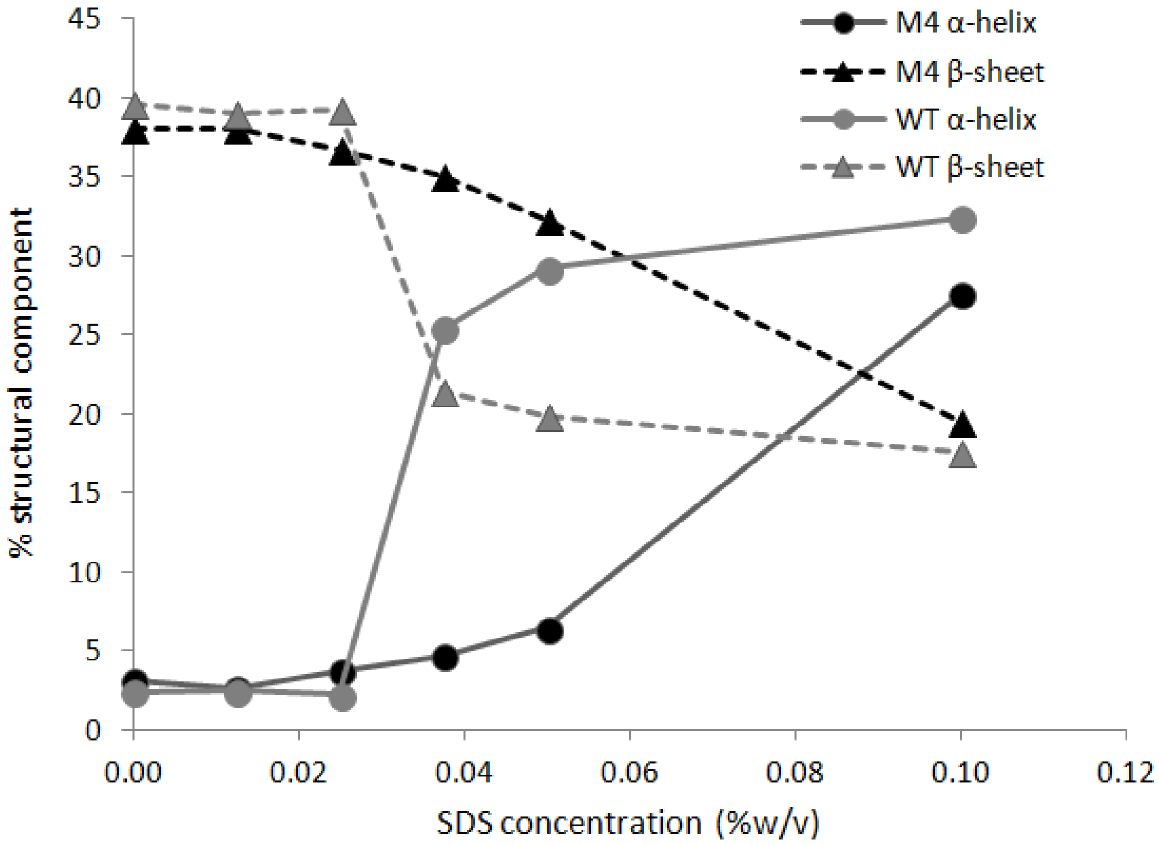

Supplementary Figure-6. **Circular dichroism analysis of WT and M4 preincubated in SDS.** Percentage α-helix and β-sheet secondary structure content in WT and M4 carbonic anhydrases incubated for 1 h with increasing concentrations of the detergent sodium dodecyl sulfate (SDS) in 10 mM Tris-HCl pH 8 added prior to circular dichroism analysis.

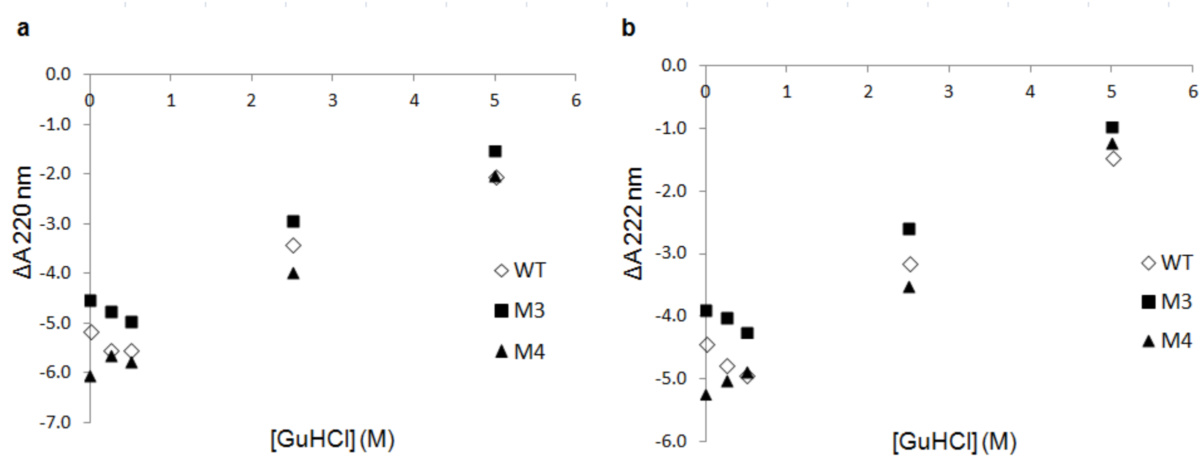

Supplementary Figure-7. Circular dichroism analysis of WT, M3 and M4. Absorbance spectra taken in the presence of guanidinium hydrochloride (0.25 - 5 M) in 10 mM Tris-HCl pH 8 as determined by circular dichroism spectroscopy at (a) 220 nm and (b) 222 nm.

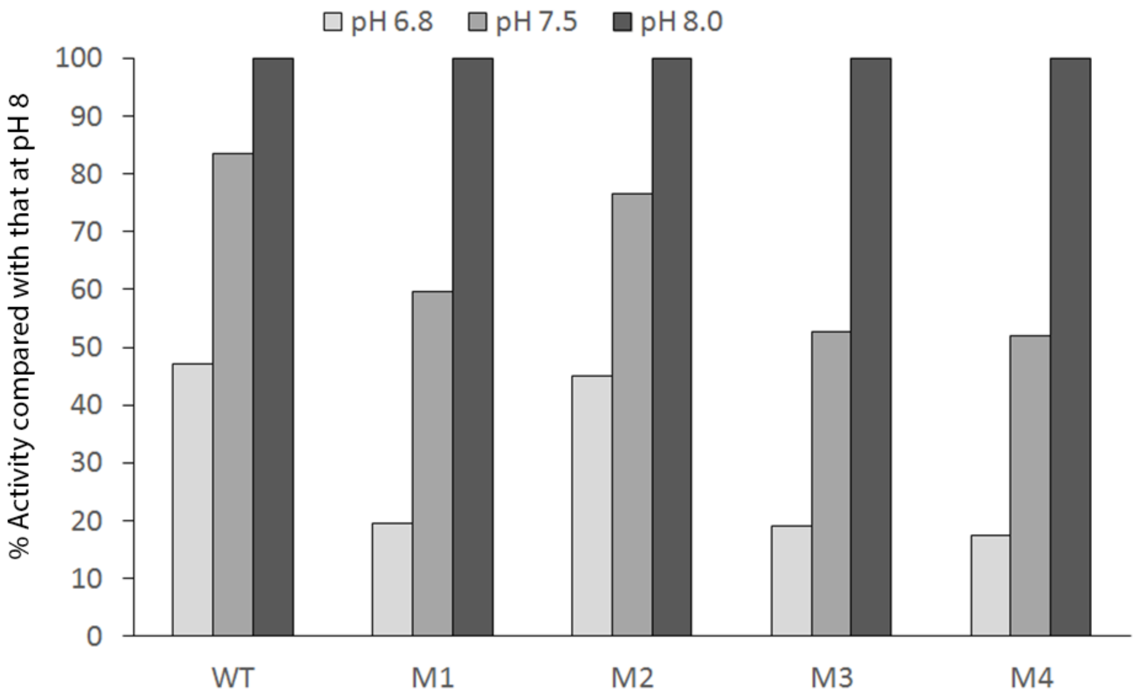

Supplementary Figure-8. **Relative 4-nitrophenyl acetate esterase activity of WT and M1 - M4**. Activity measured by spectrophotometry at the isosbestic point for 4-nitrophenol, 348 nm, at three different pH points. Activity for each enzyme is shown as percentage activity relative to the value measured at pH 8 for each enzyme.

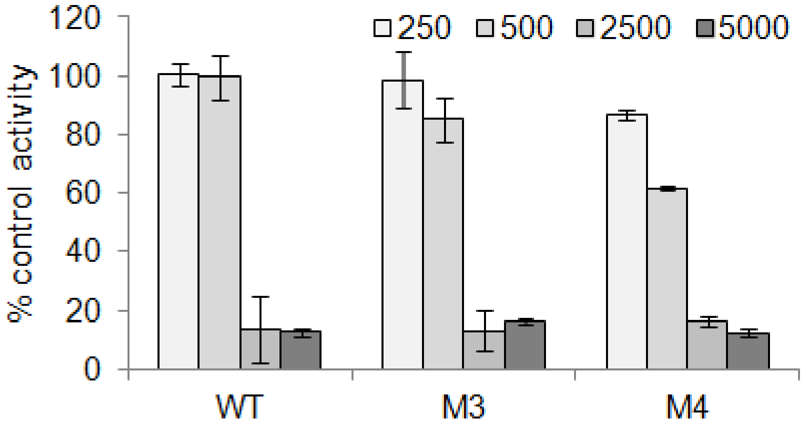

Supplementary Figure-9. **Effect of the addition of GuHCl (mM) on 4-NPA esterase activity of WT, M3 and M4**. Control activity refers to the activity of the individual enzyme in the assay buffer only.

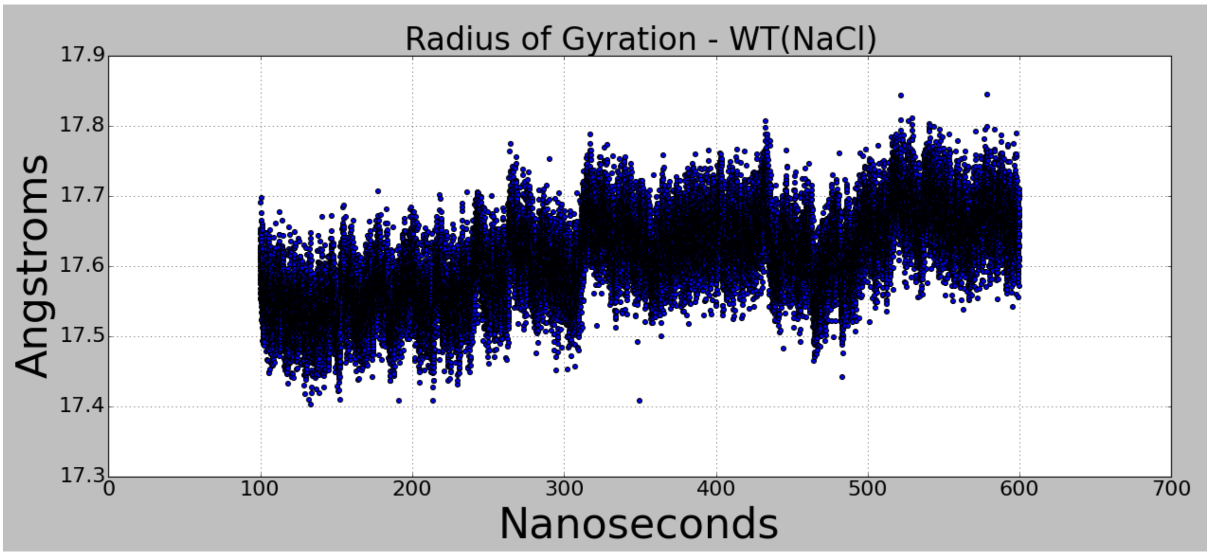
Radius of Gyration - WT(NaCl)
Angstroms
Nanoseconds
17.9
17.8
17.7
17.6
17.5
17.4
17.3
0
100
200
300
400
500
600
700

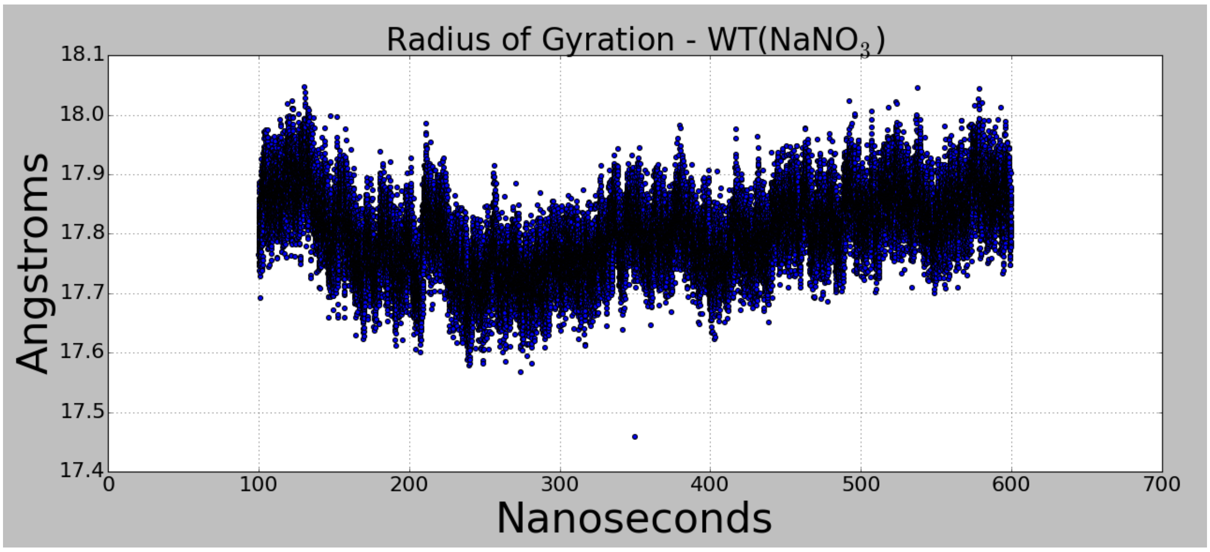
Radius of Gyration - WT(NaNO3)
Angstroms
Nanoseconds
18.1
18.0
17.9
17.8
17.7
17.6
17.5
17.4
0
100
200
300
400
500
600
700

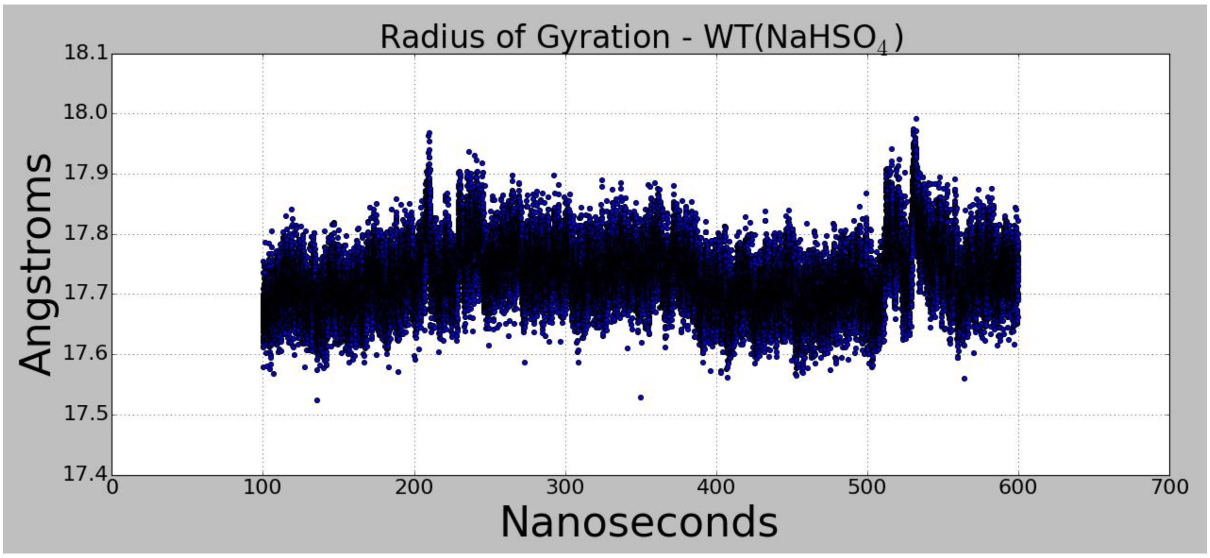
Radius of Gyration - WT(NaHSO4)
Angstroms
Nanoseconds
18.1
18.0
17.9
17.8
17.7
17.6
17.5
17.4
0
100
200
300
400
500
600
700

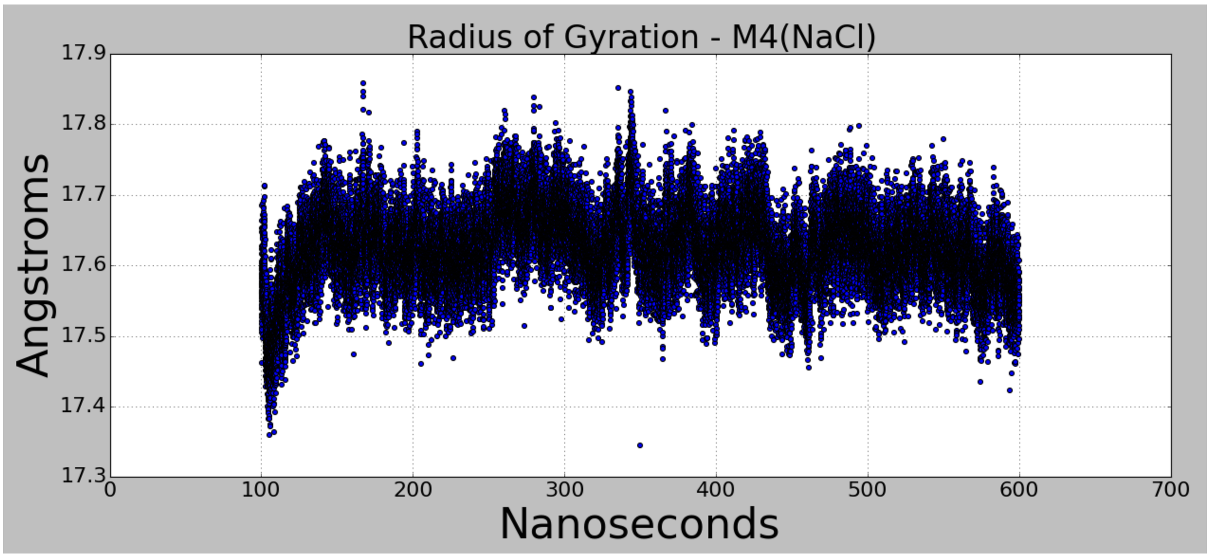
Radius of Gyration - M4(NaCl)
17.9
17.8
17.7
17.6
17.5
17.4
17.3
Angstroms
0
100
200
300
400
500
600
700
Nanoseconds

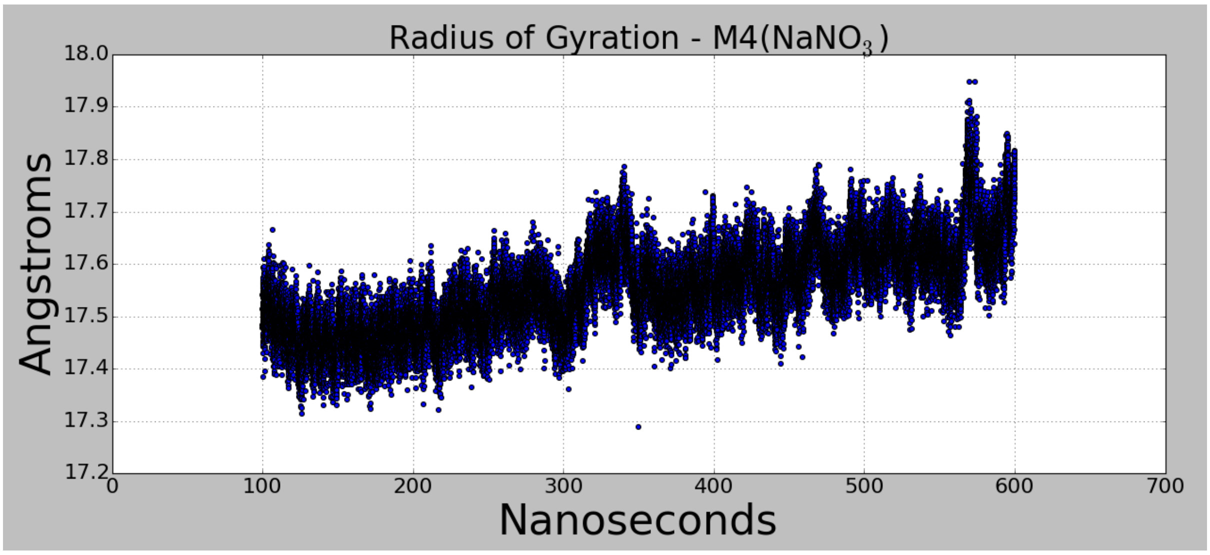
Radius of Gyration - M4(NaNO$_3$)
18.0
17.9
17.8
17.7
17.6
17.5
17.4
17.3
17.2
Angstroms
0
100
200
300
400
500
600
700
Nanoseconds

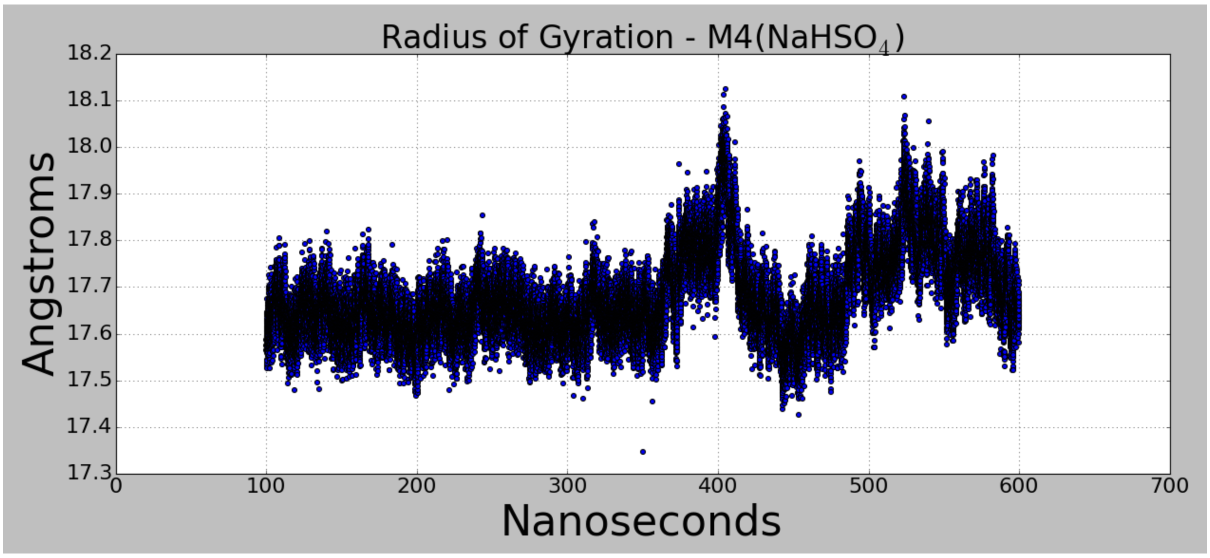
Radius of Gyration - M4(NaHSO$_4$)
18.2
18.1
18.0
17.9
17.8
17.7
17.6
17.5
17.4
17.3
Angstroms
0
100
200
300
400
500
600
700
Nanoseconds

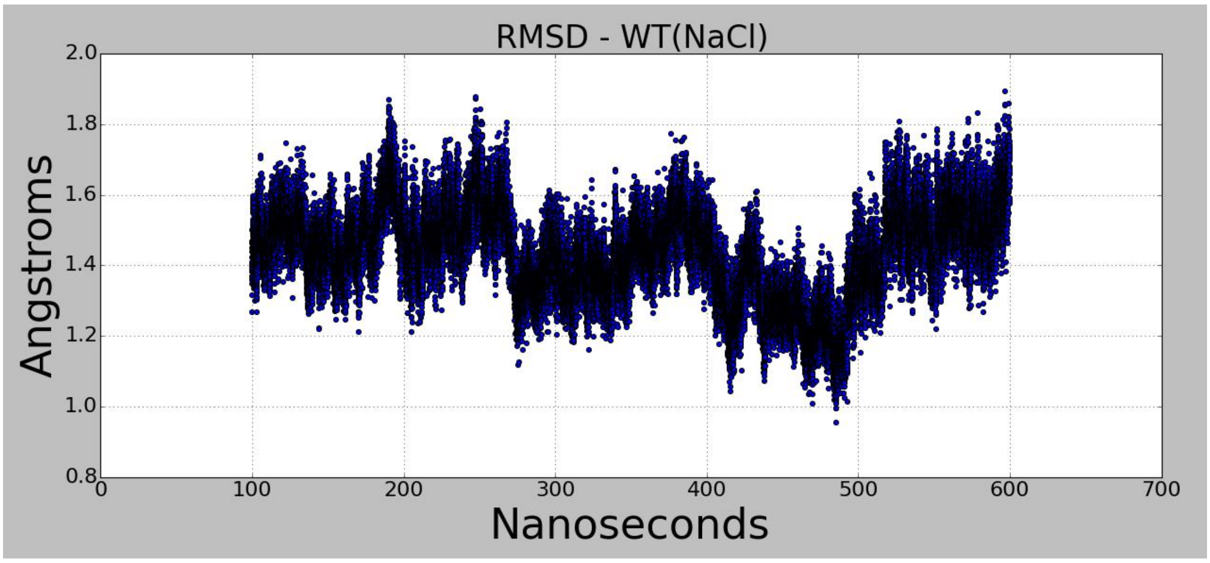
RMSD - WT(NaCl)
Angstroms
Nanoseconds
2.0
1.8
1.6
1.4
1.2
1.0
0.8
0
100
200
300
400
500
600
700

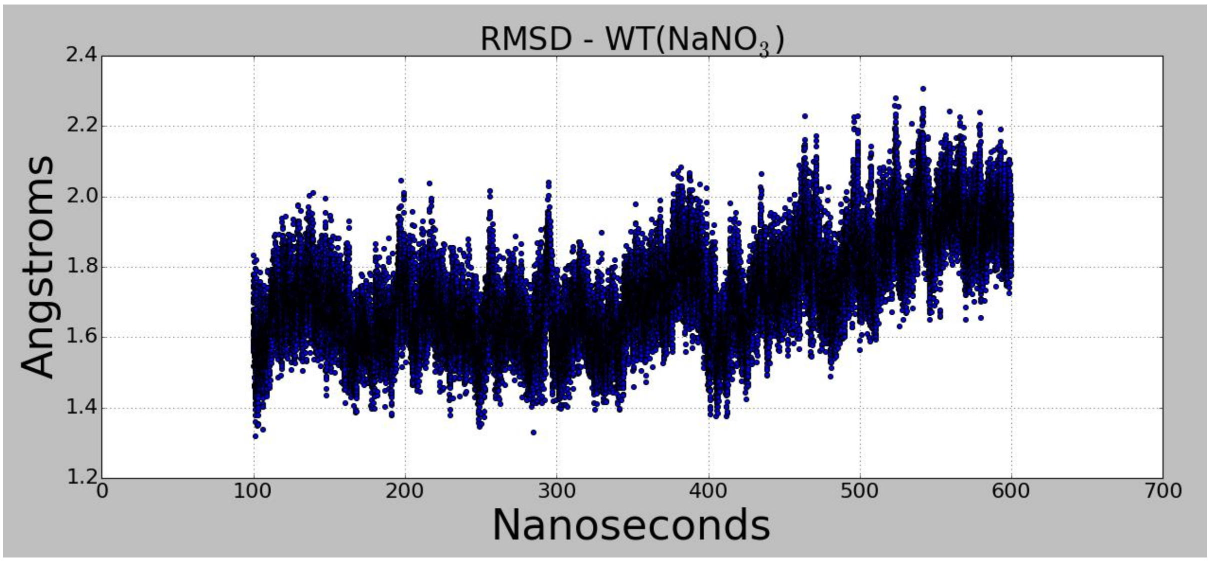
RMSD - WT($NaNO_3$)
Angstroms
Nanoseconds
2.4
2.2
2.0
1.8
1.6
1.4
1.2
0
100
200
300
400
500
600
700

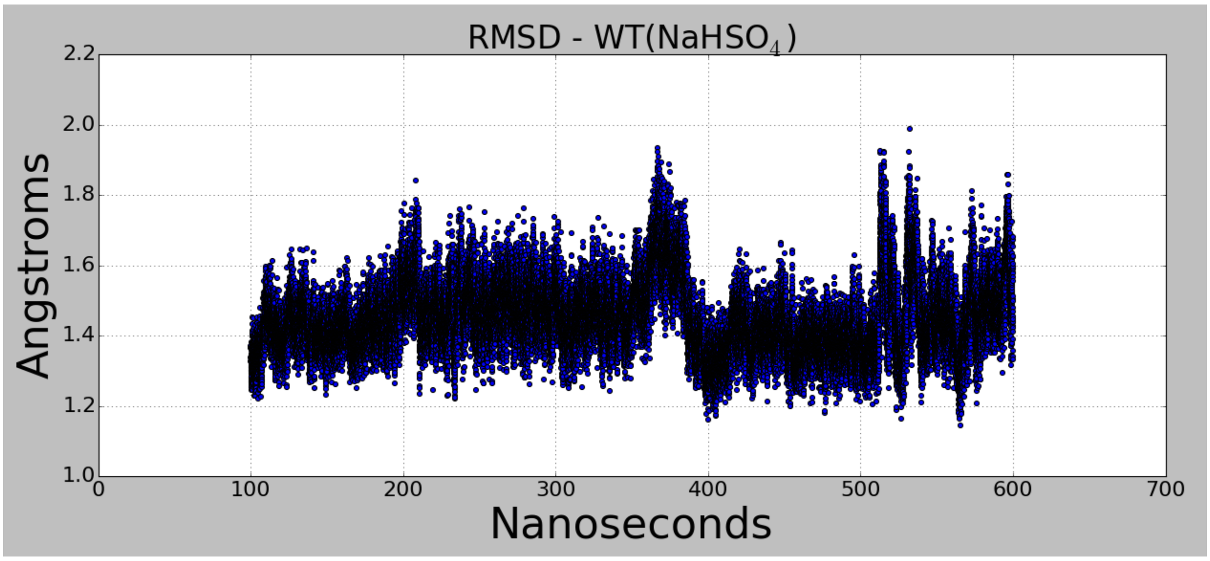
RMSD - WT($NaHSO_4$)
Angstroms
Nanoseconds
2.2
2.0
1.8
1.6
1.4
1.2
1.0
0
100
200
300
400
500
600
700

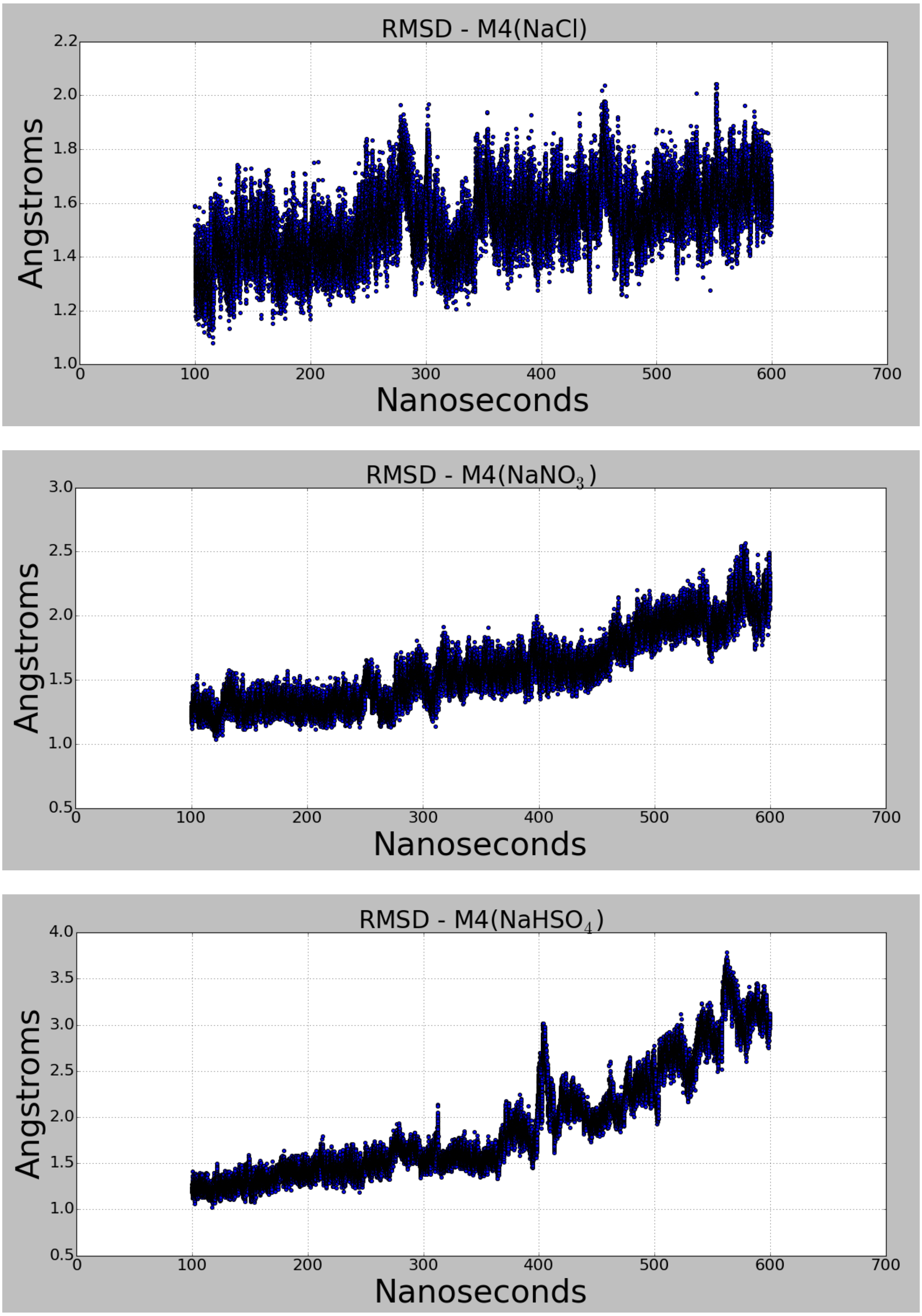

Supplementary Figure-10. **Radius of gyration and RMSD plots for the MD production periods for WT and M4**.

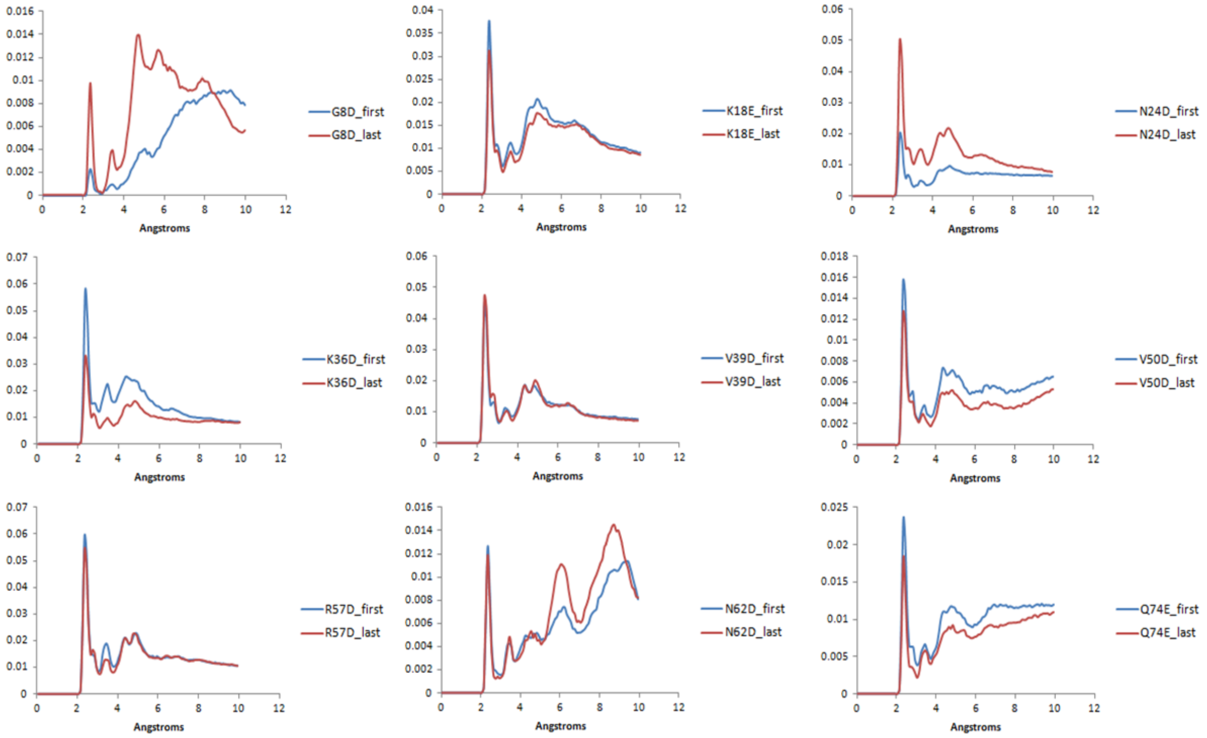
G8D_first
G8D_last
Angstroms
K18E_first
K18E_last
Angstroms
N24D_first
N24D_last
Angstroms
K36D_first
K36D_last
Angstroms
V39D_first
V39D_last
Angstroms
V50D_first
V50D_last
Angstroms
R57D_first
R57D_last
Angstroms
N62D_first
N62D_last
Angstroms
Q74E_first
Q74E_last
Angstroms

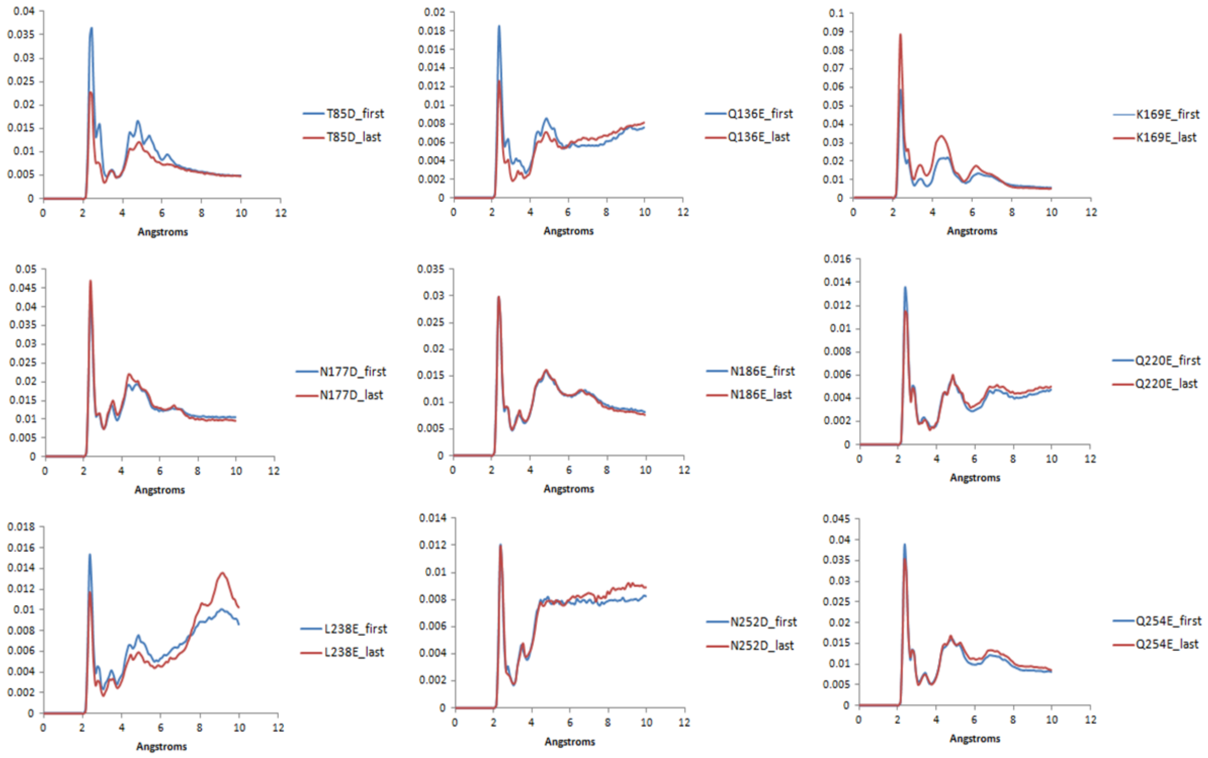

Supplementary Figure-11. **Radial distribution functions (g(r)) from the MD simulations for Na^+ and each of the mutated residues in M4 for the NaCl system**. The blue traces depict the first 250 ns of the production period and the blue traces depict the subsequent 250 ns.

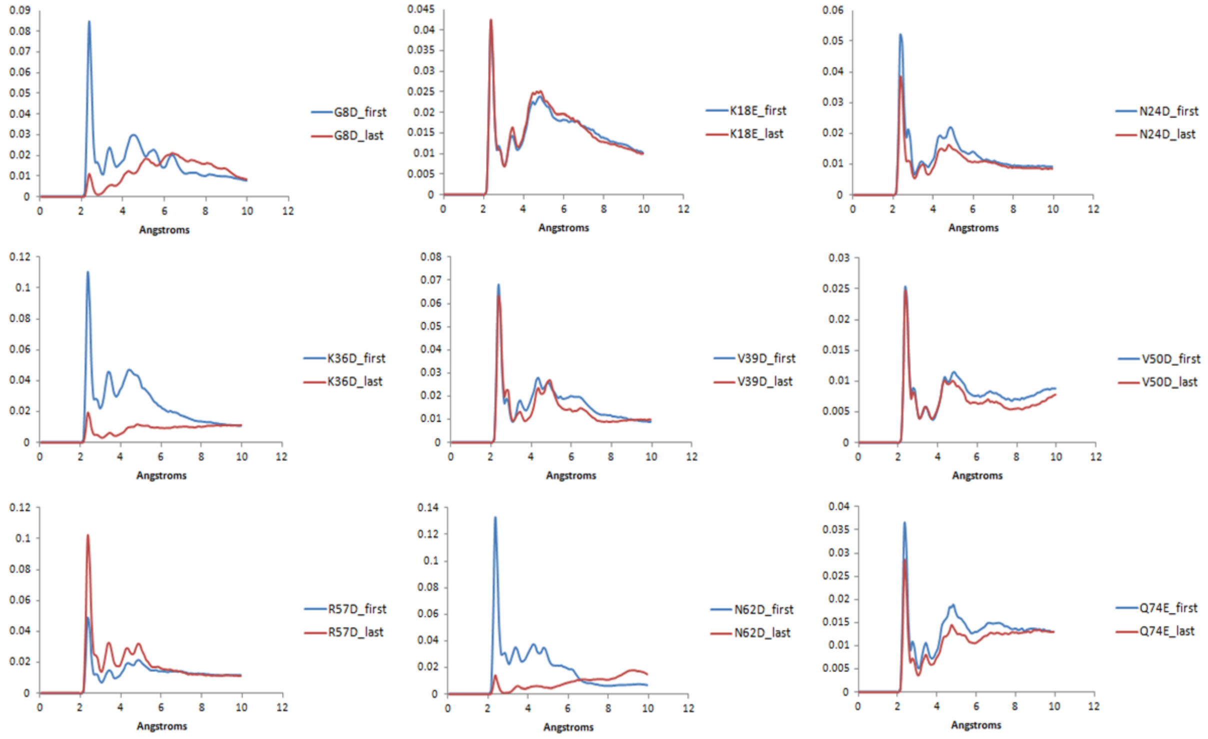

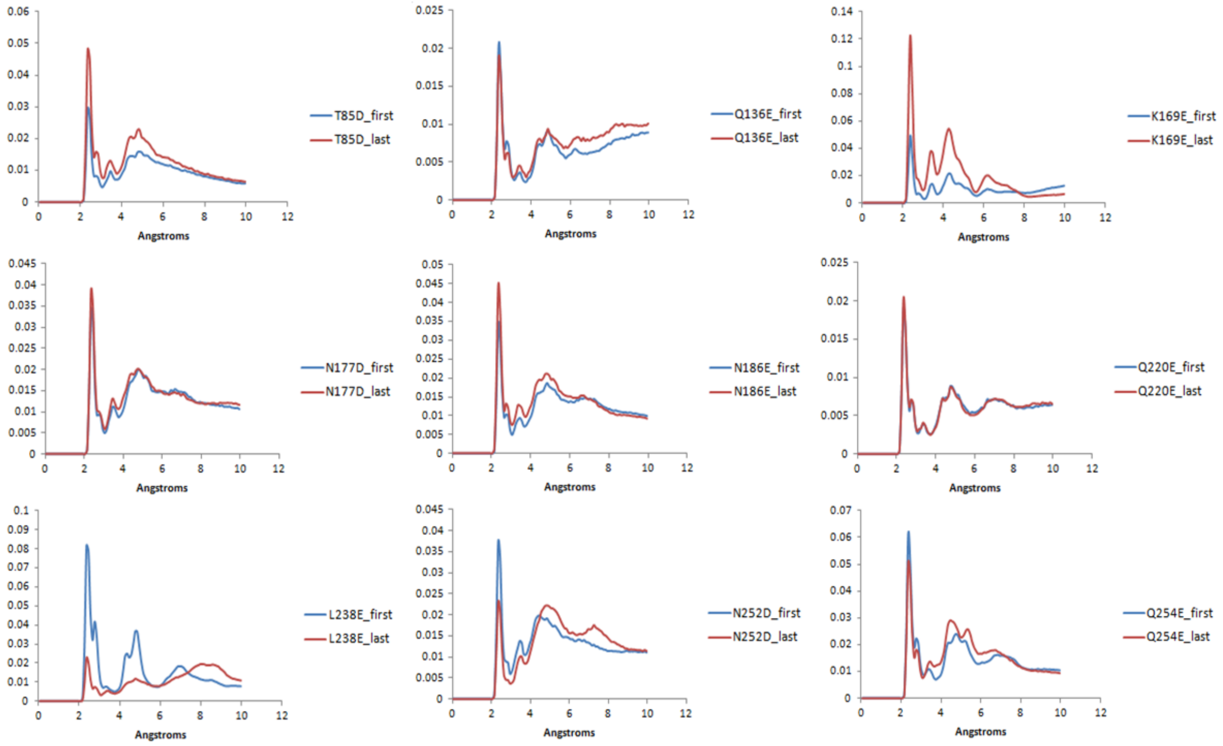

Supplementary Figure-12. **Radial distribution functions (*g*(*r*)) from the MD simulations for Na^+ and each of the mutated residues in M4 for the $NaNO_3$ system**. The blue traces depict the first 250 ns of the production period and the blue traces depict the subsequent 250 ns.

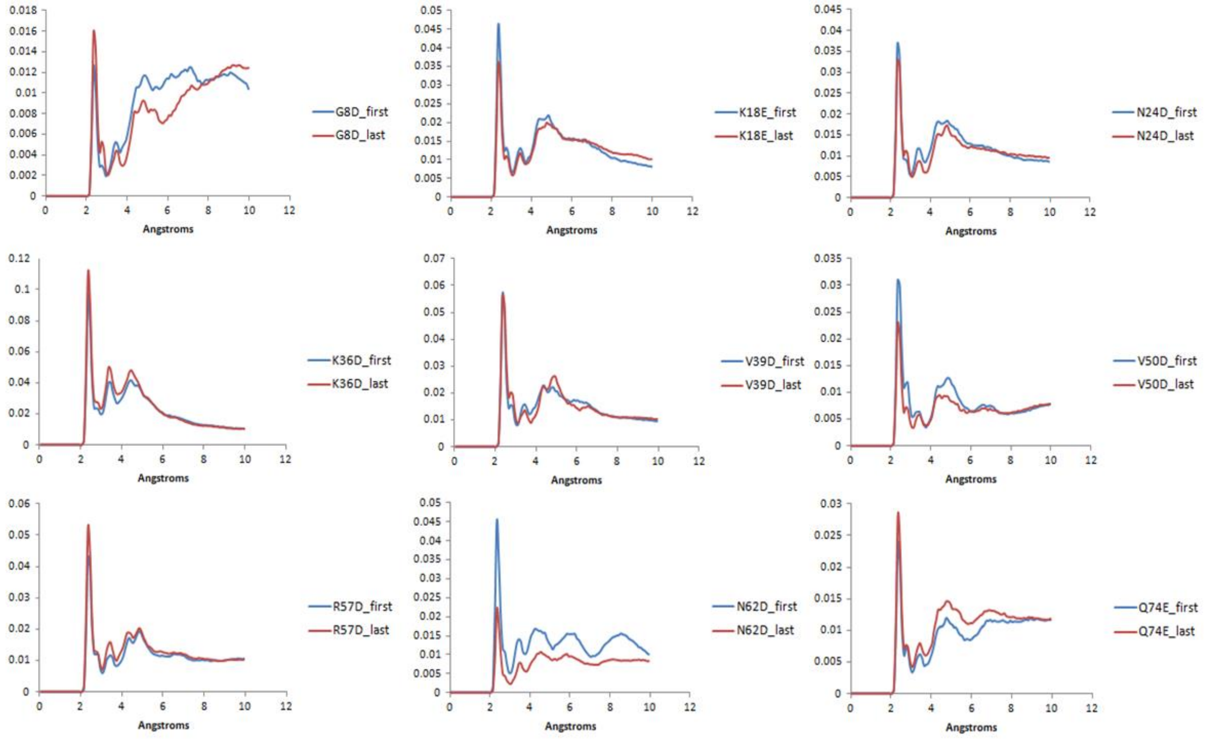

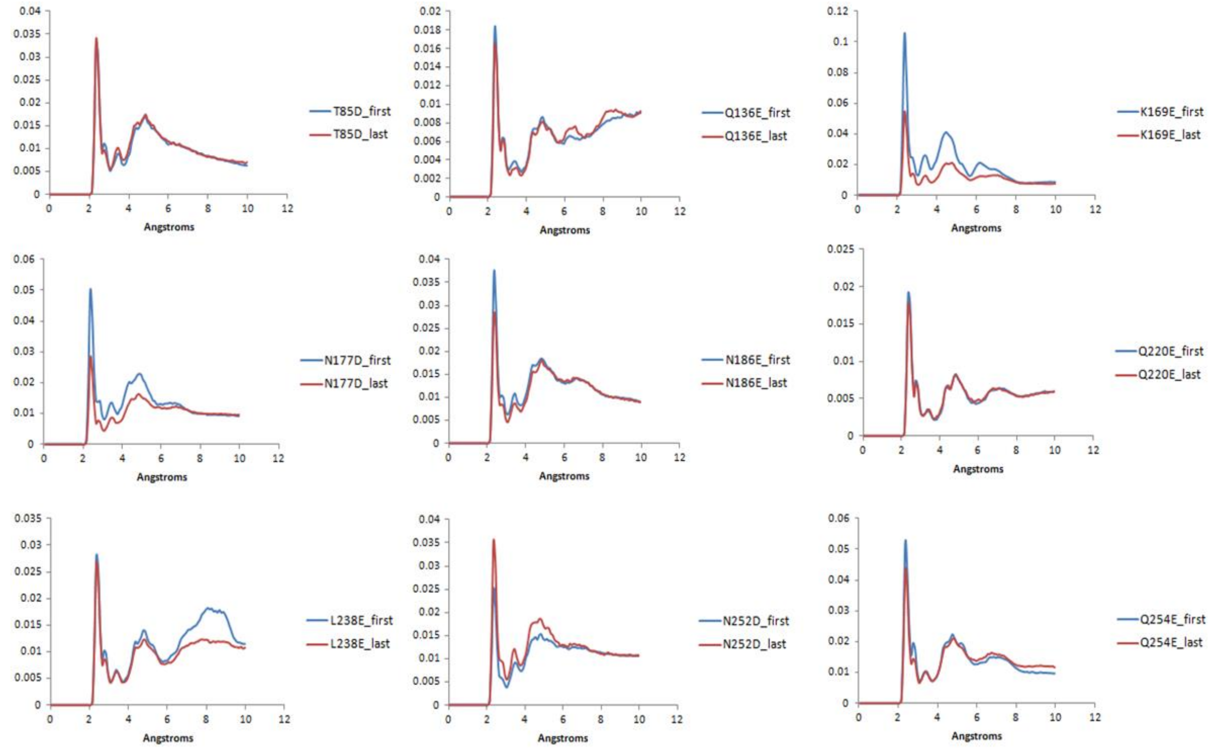

Supplementary Figure-13. **Radial distribution functions (*g*(*r*)) from the MD simulations for Na^+ and each of the mutated residues in M4 for the $NaHSO_4$ system**. The blue traces depict the first 250 ns of the production period and the blue traces depict the subsequent 250 ns.

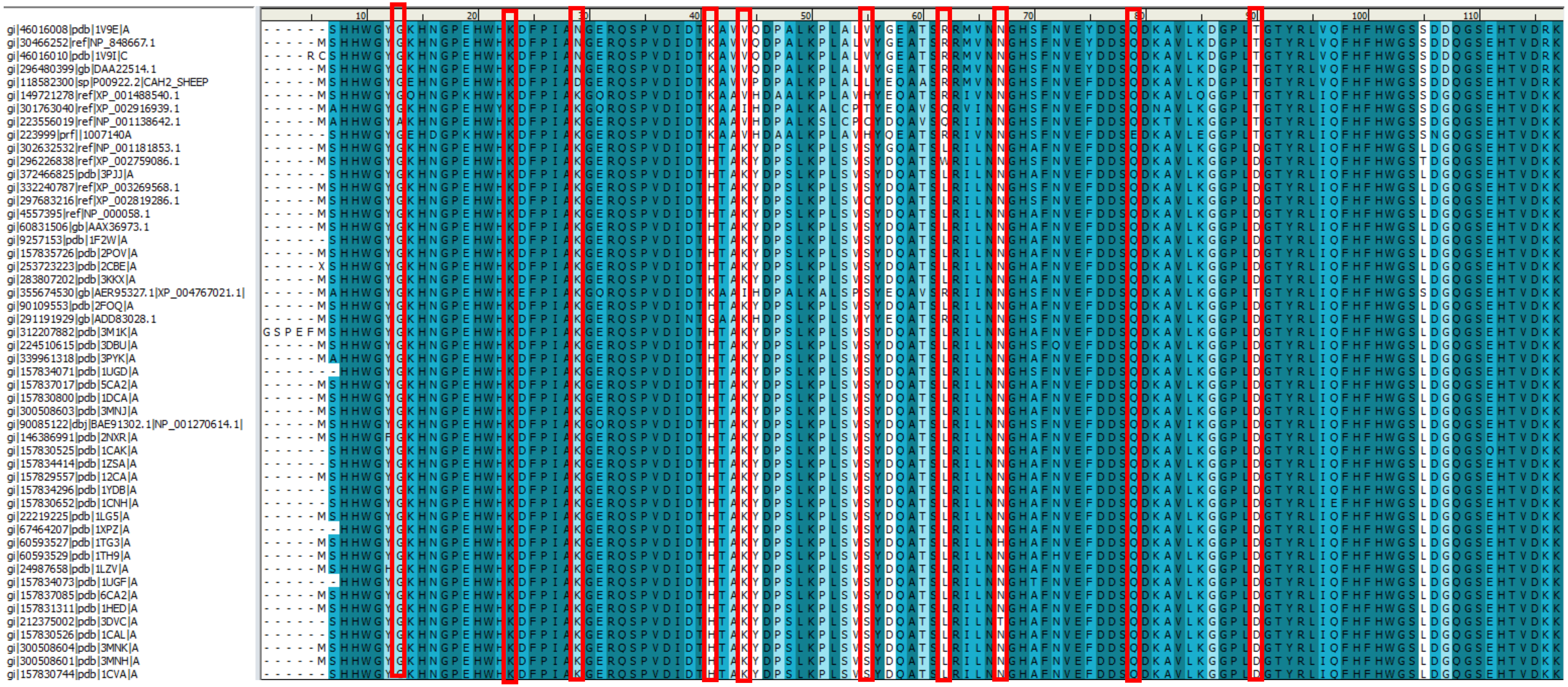

Supplementary Figure-14. ClustalW alignment of 50 amino acid sequences of closest relationship to bovine carbonic anhydrase II NP_848667.1 by BLASTp analysis. Sites within the protein considered to be tolerant of variation were selected based on their degree of substitution in related enzyme sequences and selection as a residue for mutation was made also on the basis that they were occurred on the surface of the protein. These residues are indicated in the query bovine carbonic anhydrase sequence in the top line of the alignment.

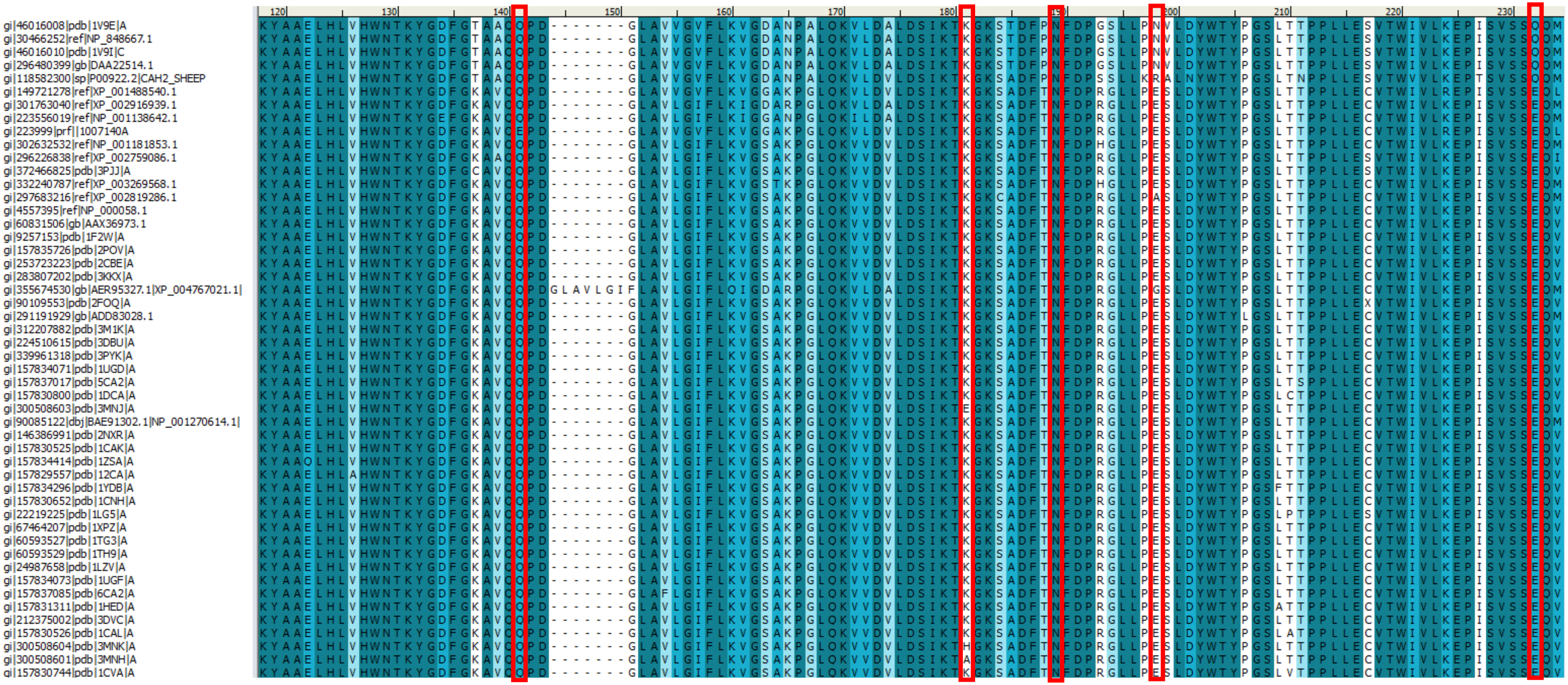

Supplementary Figure-14 (continued). **ClustalW alignment of 50 amino acid sequences of closest relationship to bovine carbonic anhydrase II NP_848667.1 by BLASTp analysis**. Sites within the protein considered to be tolerant of variation were selected based on their degree of substitution in related enzyme sequences and selection as a residue for mutation was made also on the basis that they were likely to occur on the surface of the protein. These residues are indicated in the query bovine carbonic anhydrase sequence in the top line of the alignment.

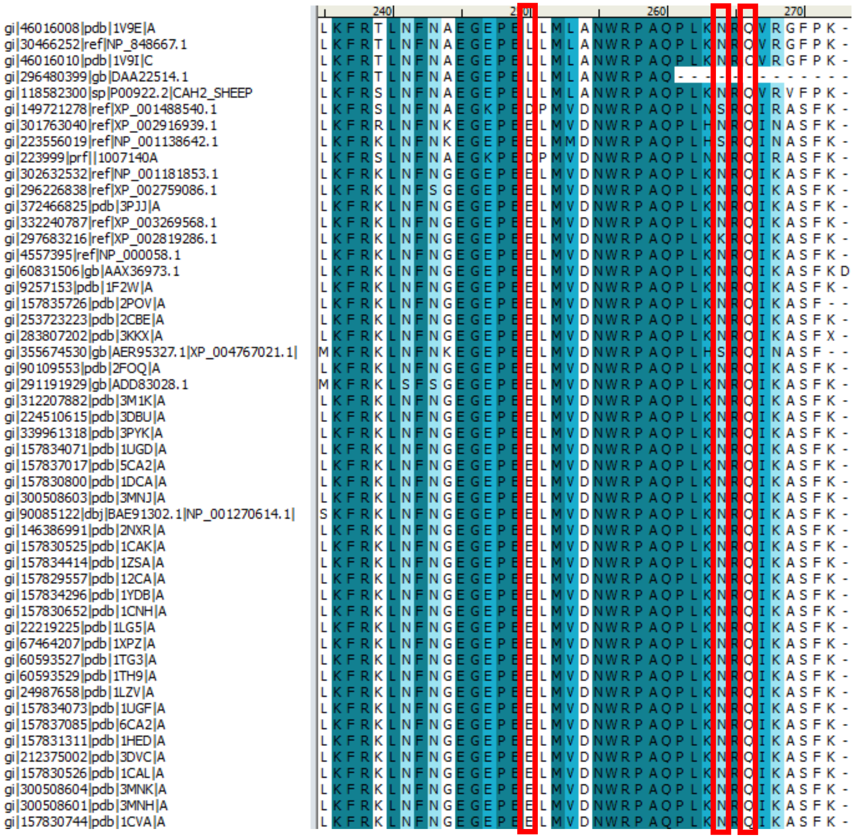

Supplementary Figure-14 (continued). **ClustalW alignment of 50 amino acid sequences of closest relationship to bovine carbonic anhydrase II NP_848667.1 by BLASTp analysis**. Sites within the protein considered to be tolerant of variation were selected based on their degree of substitution in related enzyme sequences and selection as a residue for mutation was made also on the basis that they were likely to occur on the surface of the protein. These residues are indicated in the query bovine carbonic anhydrase sequence in the top line of the alignment.

Supplementary Table 1. **Molecular weight (MW) and amino acid content of carbonic anhydrases WT and M1-M4**.

Amino acid	WT	M1	M2	M3	M4
MW	29,300	29,379	29,279	29,358	29,390
Ala	17	17	17	17	17
Cys	0	0	0	0	0
Asp	19	**23**	**23**	**27**	**29**
Glu	11	**13**	**13**	**15**	**19**
Phe	11	11	11	11	11
Gly	21	20	21	20	20
His	11	11	11	11	11
Ile	5	5	5	5	5
Lys	18	17	17	**16**	**15**
Leu	26	25	26	25	25
Met	5	5	5	5	5
Asn	13	12	**11**	**10**	**8**
Pro	19	19	19	19	19
Gln	12	11	11	**10**	**8**
Arg	9	9	8	8	8
Ser	16	16	16	16	16
Thr	14	14	14	14	13
Val	20	19	19	**18**	**18**
Trp	7	7	7	7	7
Tyr	8	8	8	8	8

Supplementary Table 2. **Summary of the IC50 values for $NaNO_3$ against the 4-NPA esterase activity of WT and M1-M4**.

4-NPA (mM)	WT	M1	M2	M3	M4
90	71	120	85	172	290
230	84	134	82	172	270
460	82	134	85	185	290
690	87	120	75	180	300
930	87	137	77	194	310
1390	81	131	85	210	270
1620	80	132	86	216	190
2000	81	129	75	193	250
Mean	81.6[a]	129.6[b]	81.2[a]	190.2[c]	271.2[d]
s.e.m.	1.7	6.4	16.9	5.8	13.4

Sodium nitrate concentrations of between 50 and 3000 mM were tested at each concentration of 4-NPA. Different lower case letters on mean values denote those that are significantly different from each other at $p<0.05$ in a Student's t test.

Supplementary Table 3. **Pertinent system parameters for each of the MD simulations**.

	WT (NaCl)	WT ($NaNO_3$)	WT ($NaHSO_4$)	M4 (NaCl)	M4 ($NaNO_3$)	M4 ($NaHSO_4$)
Volume (A3)	681,360	653,232	694,011	652,743	637,336	741,048
Total water molecules	18,059	16,594	17,327	17,087	15,949	18,554
Total atoms	58,487	54,482	57,286	55,505	52,481	60,556
Subspace overlap[a]	0.458	0.546	0.606	0.517	0.417	0.352

[a]Overlap of the eigenvectors of the backbone atoms between the first and second halves of the 500 ns production period of the MD simulations
